# Metabolomic Profiling and Immunomodulatory Activity of a Polyherbal Combination in Cyclophosphamide-Induced Immunosuppressed Mice

**DOI:** 10.3389/fphar.2021.647244

**Published:** 2022-01-03

**Authors:** Sultan Zahiruddin, Abida Parveen, Washim Khan, Mohammad Ibrahim, Muzamil Y. Want, Rabea Parveen, Sayeed Ahmad

**Affiliations:** ^1^ Bioactive Natural Product Laboratory, Department of Pharmacognosy and Phytochemistry, School of Pharmaceutical Education and Research, Jamia Hamdard, New Delhi, India; ^2^ Department of Clinical Research, School of Interdisciplinary Sciences and Technology, Jamia Hamdard, New Delhi, India; ^3^ National Center for Natural Products Research, School of Pharmacy, University of Mississippi, Oxford, MS, United States; ^4^ Roswell Park Comprehensive Cancer Center, Buffalo, NY, United States; ^5^ Department of Biosciences, Jamia Millia Islamia, New Delhi, India

**Keywords:** metabolomics, immunomodulatory activity, splenocyte, *Phyllanthus emblica*, *Withania somnifera*, *Tinospora cordifolia*

## Abstract

The study was aimed to develop a characterized polyherbal combination as an immunomodulator containing *Phyllanthus emblica* L., *Piper nigrum* L., *Withania somnifera* (L.) Dunal, and *Tinospora cordifolia* (Willd.) Miers. Through response surface methodology (RSM), the ratio of aqueous extracts of four plant materials was optimized and comprised 49.76% of *P. emblica*, 1.35% of *P. nigrum*, 5.41% of *W. somnifera*, and 43.43% of *T. cordifolia* for optimum immunomodulatory activity. The optimized combination showed antioxidant potential and contains more than 180 metabolites, out of which gallic acid, quercetin, ellagic acid, caffeic acid, kaempferitrin, and *p*-coumaric acid are some common and significant metabolites found in plant extracts and in polyherbal combination. Treatment with the polyherbal combination of different doses in cyclophosphamide-induced immunosuppressed mice significantly (p < 0.01) enhanced the subsets of immune cells such as natural killer (NK) cells (60%), B cells (18%), CD4 cells (14%), and CD8 cells (7%). The characterized polyherbal combination exhibited potent immunomodulatory activity, which can be further explored clinically for its therapeutic applicability.

## Introduction

Herbs are in increasing demand in developed and developing countries for primary health care. Plant-based products have been extensively exploited as an essential source for drugs because of their biological activities and higher safety margins. Immunomodulation using medicinal plants can provide a substitute for conventional therapy for a variety of diseases, especially when the host’s defense mechanism has to be generated under the conditions of an impaired immune response (Ganju et al., 2003). People, every year, turn to herbal medicine because they believe that plant remedies are free from undesirable side effects. It is also known that herbs have been used in traditional systems of medicine as an immunomodulator for a decade (Kumar et al., 2012; Nagoba and Davane, 2018). Plant extracts have been proven for their activity in boosting the humoral (Rehman et al., 1999) as well as cell-mediated immunity against viruses, bacteria, fungi, and cancer (Sarvanandaa et al., 2018). In the Indian traditional system of medicine, several food-grade plants have been used as immunomodulators, and most of them were proven individually for their immune-enhancing effect. The bioactive constituents of these plants that are responsible for the activity were also determined. In recent years, many herbs from the Indian system of medicine that modulate the immune system of the body were scientifically validated. Some herbs such as *Phyllanthus emblica* L. (family: Phyllanthaceae) (Liu et al., 2012), *Piper nigrum* L. (family: Piperaceae) (Majdalawieh and Carr, 2010), *Tinospora cordifolia* (Willd.) Miers (family: Menispermaceae) (Mathew and Kuttan, 1999), *Withania somnifera* (L.) Dunal (family: Solanaceae) (Davis and Kuttan, 2000), *Curcuma longa* L. (family: Zingiberaceae) (Kim et al., 2014), *Ocimum sanctum* L. (family: Lamiaceae) (Mondal et al., 2011), *Azadirachta indica* A. Juss. (family: Meliaceae) (van der Nat et al., 1987), *Achilla millefolium* L. (family: Asteraceae) (Mohammed Al-Ezzy et al., 2018), and many more are the reported herbs exhibiting excellent immunomodulatory activity. Based on our preliminary study, we focused only on the first four plants, namely, *P. emblica*, *P. nigrum*, *T. cordifolia*, and *W. somnifera*.

*P. emblica*, Indian gooseberry, is an essential medicinal plant in the Indian traditional system of medicine and is used as a dietary combination. It has various properties that can combat age-related illnesses such as cancer, cardiac diseases, renal failure, immune deficiency, arthritis, cataracts, and wrinkling of the skin (Mathai et al., 2015). *T. cordifolia* is of great interest to scientists around the world due to its anti-inflammatory, antiarthritic, antioxidant, antiallergic, antistress, antileprotic, antimalarial, hepatoprotective, immunomodulatory activities (More and Pai, 2011; Alsuhaibani and Khan, 2017). The modern medical literature, as well as traditional medical literature, reports many potential health benefits of *W. somnifera* in stress, neurotoxicity, and immune disorders (Singh et al., 2011; Zahiruddin et al., 2020). *P. nigrum* is used extensively as a spice around the world and in traditional medicine as an antioxidant, antimicrobial, anticarcinogenic, anti-inflammatory, and gastroprotective drug (Parveen et al., 2015; Bui et al., 2019). These four selected plant extracts were further subjected to optimization of the ratio by response surface methodology (RSM) to develop a polyherbal combination.

Across the globe, several herbal formulations are being sold claiming to be immunomodulators. Vivartana, Chyawanprash, Brahma Rasayana, IM-133, and Septilin are the few formulations used in the Indian traditional system of medicine, which have been experimentally proven as immunomodulators (Gnanasekaran et al., 2015). But these formulations have not been fully explored scientifically, and the dose and frequency of these formulations are much higher than those of modern medicines. In the present study, we selected eight traditionally used plants according to the literature reported for immunomodulatory activity, and out of eight, only four named *P. emblica*, *P. nigrum*, *T. cordifolia*, and *W. somnifera* were found to be the best immunomodulators in our preliminary studies. These four selected plants were used to develop a polyherbal combination. The proposed study aims to develop a polyherbal combination as an immunomodulator, which can be used for the management of immunity for the prevention of various bacterial and viral infections. This herbal combination has been chromatographically characterized and scientifically validated through *in vitro* and *in vivo* experiments.

## Methodology

### Collection of Plant Materials

The fruits of *P. emblica*, *P. nigrum*, stem of *T. cordifolia*, and roots of *W. somnifera* were purchased from Universal Biotech, Farash Khana, Delhi, India. The voucher specimens (*P. emblica*-BNPL/JH/Ph.D/12/17/01, *T. cordifolia*-BNPL/JH/Ph.D/12/17/02, *W. somnifera-*BNPL/JH/Ph.D/12/17/03, and *P. nigrum*-BNPL/JH/Ph.D/12/17/04) of each plant material have been deposited in the Bioactive Natural Product Laboratory (BNPL), Jamia Hamdard, New Delhi, India, for future reference.

### Preparation of the Extract

Accurately weighed 150 g of powder of each plant material were divided into three equal parts. These were macerated overnight using water, ethanol, and 50% ethanol to form aqueous extract (AE), ethanolic extract (EE), and hydroethanolic extract (HEE), respectively. The EE and HEE were further refluxed for 3 h after maceration. All three extracts were filtered, and the filtrate was evaporated to dryness under reduced pressure. The extractive values and percentage yields of different extracts were calculated and stored at 4°C for further analysis.

### Quantitative Analysis of Specific Marker Compounds From Raw Extracts

Gallic acid, piperine, berberine, and withaferin-A were selected as specific marker compounds for fruits of *P. emblica*, *P. nigrum*, stem of *T. cordifolia*, and roots of *W. somnifera*, respectively (Chatterjee et al., 2012; Gorgani et al., 2017; Neag et al., 2018; Tiwari et al., 2018). These markers were quantified using high-performance thin-layer chromatography (HPTLC). Ten milligrams of each extract were dissolved separately in high-performance liquid chromatography (HPLC)-grade methanol to get 10-mg/ml solutions. The stock solutions of standard gallic acid, berberine, piperine, and withaferin-A (Sigma Aldrich, India) were prepared in HPLC-grade methanol to get a concentration of 500 μg/ml. The prepared samples were filtered using a 0.2-μm polytetrafluoroethylene (PTFE) membrane filter before HPTLC analysis. The prepared extracts and standards were applied separately on Silica gel 60 F254 precoated TLC plates, 10 cm × 10 cm (Merck, Germany), with the help of a Camag Linomat (CAMAG, Switzerland) applicator with nitrogen flow providing a delivery speed of 120 nl/s from the syringe. Toluene:ethyl acetate:formic acid (5:4:1; v/v/v) were used as developing solvents for gallic acid, piperine, and withaferin-A. For berberine, *n*-butanol:water:acetic acid (4:5:1; v/v/v) was used as a developing solvent. Plates were developed horizontally in a CAMAG twin trough glass chamber (10 cm × 10 cm), which was presaturated with the mobile phase for 30 min. The developed plates were air-dried and scanned by CAMAG TLC densitometric scanner III operated by WinCATs software. Gallic acid and piperine were scanned at 254 nm, while berberine was scanned at 366 nm. For quantitative analysis of withaferin-A, the air-dried plate was derivatized by dipping in 5% anisaldehyde sulfuric acid solution and scanned at 540 nm.

### Response Surface Methodology for Optimization of Extract Ratio

RSM was used to optimize the ratio of four extracts, which is a collection of statistical techniques to design experiments, build models, evaluate the effect of factors, and find the optimum conditions. Four extracts at three different concentration levels were combined by Box–Behnken response surface design (BBD), where pinocytic activity in macrophages and splenocyte proliferation assay were taken as the response of different combinations. Different concentrations of the aqueous extract of *P. emblica*, *P. nigrum*, *W. somnifera*, and *T. cordifolia* were selected to optimize the effective concentration. The dose of each selected plant extract was calculated from its extractive values, which is equivalent to the dose mentioned in Ayurveda Pharmacopoeia of the respective plant materials. The selected concentrations for *P. emblica* were 100 mg (−1), 550 mg (0), and 1,000 mg (+1); concentrations of *P. nigrum* were 6 mg (−1), 33 mg (0), and 60 mg (+1); concentrations of *W. somnifera* were 10 mg (−1), 455 mg (0), and 900 mg (+1); and the concentration levels for *T. cordifolia* were 6 mg (−1), 333 mg (0), and 660 mg (+1). A total of 29 experimental combinations with three central points were generated through BBD using Design Expert 8.0.1.7 software (Stat-Ease Inc., USA), and all these combinations were tested for their pinocytic activity in macrophages and splenocyte proliferation assay (Table 1). The point prediction tool of the software determined an optimum value of the factors for the maximum *in vitro* immunomodulatory activity.

**TABLE 1:** Four-variable Box–Bhenken design with experimental and predicted values of pinocytic and splenocyte proliferation of different combinations of aqueous extracts.

Run no.	Concentration of aqueous extract (mg)				Pinocytic activity		Splenocyte proliferation	
	A	B	C	D	Predicted	Actual	Predicted	Actual
1	100	6	455	333	0.028	0.058	0.027	0.012
2	1,000	6	455	333	0.027	0.019	0.180	0.220
3	100	60	455	333	0.017	0.004	0.055	0.110
4	1,000	60	455	333	0.180	0.150	0.031	0.081
5	550	33	10	6	0.004	0.033	0.260	0.240
6	550	33	900	6	0.059	0.051	0.240	0.270
7	550	33	10	660	0.078	0.076	0.240	0.320
8	550	33	900	660	0.110	0.067	0.047	0.160
9	100	33	455	6	0.022	0.001	0.140	0.077
10	1,000	33	455	6	0.067	0.083	0.240	0.260
11	100	33	455	660	0.055	0.053	0.240	0.160
12	1,000	33	455	660	0.055	0.091	0.140	0.150
13	550	6	10	333	0.059	0.070	0.280	0.270
14	550	60	10	333	0.029	0.040	0.220	0.180
15	550	6	900	333	0.005	0.007	0.140	0.130
16	550	60	900	333	0.110	0.110	0.240	0.190
17	100	33	10	333	0.028	0.017	0.052	0.120
18	1,000	33	10	333	0.170	0.130	0.320	0.250
19	100	33	900	333	0.062	0.071	0.074	0.100
20	1,000	33	900	333	0.039	0.048	0.260	0.150
21	550	6	455	6	0.020	0.008	0.160	0.190
22	550	60	455	6	0.070	0.077	0.230	0.220
23	550	6	455	660	0.063	0.070	0.270	0.230
24	550	60	455	660	0.048	0.074	0.230	0.160
25	550	33	455	333	0.120	0.057	0.260	0.230
26	550	33	455	333	0.071	0.057	0.230	0.230
27	550	33	455	333	0.012	0.057	0.240	0.230
28	550	33	455	333	0.100	0.057	0.290	0.230
29	550	33	455	333	0.005	0.057	0.120	0.230

A: *P. emblica*; B: *P. nigrum*; C: *W. somnifera*; D: *T. cordifolia*. Levels for *P. emblica* concentration are 100 mg (−1), 550 mg (0), and 1,000 mg (+1); for *P. nigrum* are 6 mg (−1), 33 mg (0), and 60 mg (+1); for *W. somnifera* are 10 mg (−1), 455 mg (0), and 900 mg (+1); and for *T. cordifolia* are 6 mg (−1), 333 mg (0), and 660 mg (+1).

### Metabolomic Profiling of Extracts and the Developed Polyherbal Combination by Ultra-Performance Liquid Chromatography-Mass Spectrometry

The optimized polyherbal combination and four aqueous extracts used to make the polyherbal combination were dissolved separately in LC-MS-grade methanol and filtered through a 0.2-µm membrane filter. The filtered solutions were diluted in a ratio of 1:5 (v/v) using methanol. High-throughput profiling of metabolites was carried out by ultra-performance liquid chromatography-mass spectrometry (UPLC-MS) (Zahiruddin et al., 2017). The UPLC was performed on a Water’s ACQUITY UPLC^(^™^)^ system (Serial No. F09 UPB 920M; Model code # UPB, Waters Corp., MA, USA) equipped with a binary solvent delivery system, an auto-sampler, column manager, and a tunable MS detector (Serial No. JAA 272; Synapt; Waters, Manchester, UK) installed and controlled by Mass Lynx V 4.1 (Waters, USA). Data acquisition has been carried out in positive modes. Chromatography was performed using acetonitrile (A) and water (B) as the mobile phase on a monolithic capillary silica-based C18 column [ACQUITY UPLC(R) BEH C18, 1.7 µm, 2.1 × 100 mm], with the pre-column split ratio of 1:5 min at ambient temperature. Chromatographic separation was achieved by gradient elution mode (initially, 10% A; 0–5 min 40% A; 5–10 min 60% A; 10–13 min, 90% A; 13–15 min, 100% A; 15–16 min 10% A), and the total run time was 16 min. The flow rate of the nebulizer gas was set at 10 μl/min; for the cone gas, set to 50 L/h, and the source temperature was set at 100°C. The cone and capillary voltages were set to 40.0 and 3.0 kV, respectively. For collision, argon was employed at a pressure of 5.3 × 10^–5^ Torr. The accurate mass and composition for the precursor ions and the fragment ions were calculated using the Mass Lynx V 4.1 software incorporated in the instrument.

Raw data obtained from UPLC-MS analysis were processed in Progenies Software (Waters, USA). By using this software, the maximum number of mass peaks was detected, which were further identified through the library based on their molecular weight. Chromatographically separated and identified metabolites were matched with the plant’s native metabolites. All detected metabolites were processed in XLStat software for statistical differentiation between the plants used for the development of polyherbal combination. A metabolite-based comparison was performed in different extracts to identify the major metabolites present in the specific plant extract.

### Characterization of Polyherbal Combination

#### Determination of Total Phenol and Flavonoid Contents

The total phenol content (TPC) and the total flavonoid content (TFC) of the polyherbal combination were determined using the Folin–Ciocalteu (FC) and aluminum chloride methods, respectively (Khan et al., 2017). The optimized combination was dissolved in methanol to prepare 5 mg/ml. For the determination of the total phenolic content (TPC), 500 µl of methanolic solution (5 mg/ml) was thoroughly mixed with 2.5 ml FC and 2.5 ml of sodium carbonate (7.5% w/v). The mixture was incubated for 30 min at room temperature, and after incubation, absorbance was measured at 765 nm. For the determination of the TFC, 0.1 ml of aluminum chloride (10% w/v) and 0.1 ml of potassium acetate (0.1 mM) were added in 500 µl of sample solution (5 mg/ml). The resulting mixture solution was kept at room temperature for 30 min, and absorbance was measured at 415 nm. TPC and TFC were calculated from the calibration curve of gallic acid and rutin (Sigma Aldrich, India), respectively, and the results were expressed as milligrams gallic acid and rutin, respectively, equivalent per gram weight of the polyherbal combination.

#### Antioxidant Activity of the Optimized Combination

The antioxidant potential of the developed polyherbal combination was determined by measuring the scavenging potential of 2,2-diphenyl-1-picrylhydrazyl (DPPH; SRL, India). One milliliter of freshly prepared DPPH solution (0.3 mM in methanol) was mixed with 1.0 ml of optimized combination dissolved in water. The resulting mixture was kept at room temperature in the dark for 25 min, and after incubation, absorbance was recorded at 515 nm. Different concentrations of optimized combinations were used to determine DPPH radical scavenging activity (Liu et al., 2009). The ability of the sample to scavenge the DPPH radicals was calculated using the following formula:

$$\text{DPPH}\;\text{radical}\;\text{scavenging}\;\text{effect}=\left({\text{A}}_{\text{control}}{-\text{A}}_{\text{sample}}\right)\text{×}100/{\text{A}}_{\text{control}}$$

where A_control_ is the absorbance of the DPPH solution without polyherbal combination; A_sample_ is the absorbance of the sample with DPPH solution.

To determine the reducing power, 500 µl of an aqueous solution of the optimized combination was thoroughly mixed with 2.5 ml of potassium ferricyanide (1% w/w) and 2.5 ml of phosphate buffer (pH 6.6), and the mixture was incubated for 30 min in a water bath at 50°C. After incubation, the mixture was cooled, and 2.5 ml of trichloroacetic acid (10% w/v in water) was added. Furthermore, it was centrifuged and the supernatant was diluted with an equal amount of deionized water, and freshly prepared 0.5 ml of ferric chloride (0.1% w/w) was added. The mixture was thoroughly mixed, and its absorbance was measured at 700 nm (Bhalodia et al., 2013). For control, water was used in place of an optimized combination, and the reducing power was compared with the control. For both assays, ascorbic acid was used as a positive control.

### *In Vitro* Immunomodulatory Activity of the Developed Polyherbal Combination

#### Animals

BALB/c mice (6 weeks old, 25 ± 5 g) were provided by Central Animal House Facility, Jamia Hamdard (Registration No. 173/GO/RE/S/2000/CPCSEA). Before initiation of the experiment, all experimental protocols were approved by the Institutional Animal Ethics Committee, and the experiments were strictly carried out according to the guidelines of the committee (Animal Approval Number 1551). The animals were housed in a polypropylene cage and placed in the experimental room, where they were allowed to acclimatize for a week before the experiment. An air conditioning unit (with 10% air exchange per hour) was maintained along with a relative humidity of 50 ± 10 RH, 12/12 h light–dark cycle, and a temperature of 25°C ± 2°C was kept in the animal house facility throughout the experimental period.

#### Splenocyte Proliferation Assay

BALB/c mice were euthanized, and the spleen was removed aseptically for isolation of splenocytes. The spleen was further homogenized using sterile phosphate-buffered saline (PBS) by passing it through a mesh (0.4 microns) and centrifuged at 300 × g for 5 min. Red blood cells (RBCs) were lysed from the cell pellet by adding 500 µl of lysis buffer (Tris-HCl-NH_4_Cl, pH 7.2). The reaction was stopped by adding Roswell Park Memorial Institute (RPMI) medium (RPMI-1640, Millipore Sigma Aldrich, India) and washed two times to remove any debris. The cell pellet was resuspended in RPMI medium to get 3.0 × 10^6^ cells/ml, and 100 µl of cell suspension was seeded per well in a 96-well plate. Here, 25 µl of mitogen concanavalin A (Con A; 2.0 μg/ml) was added, and 25 µl of the polyherbal combination was added to the cells per well containing splenocytes and incubated at 37°C in a CO_2_ incubator for 72 h. After incubation, 20 µl of tetrazolium dye 3-(4,5-dimethylthiazol-2-yl)-2,5-diphenyltetrazolium bromide (MTT) were added and incubated at 37°C, 5% CO_2_ for another 6 h. Furthermore, the tetrazolium crystals formed in live cells were dissolved after 6 h of incubation, and cell proliferation was measured at 490 nm using a microplate reader (Shi and Fu, 2011). The media treated with polyherbal combination was used as a negative control, and spleen cell treated with levamisole was used as the positive control.

#### Pinocytic Activity Assay

One milliliter of thioglycolate (1.0 mg/ml in PBS) was injected intraperitoneally (i.p.) in 2 mice, and peritoneal macrophages were isolated after 48 h by injecting PBS into the peritoneal cavity. Isolated macrophages were washed twice with media and resuspended at 1.0 × 10^6^ cells/ml in RPMI medium containing 10% fetal bovine serum (FBS). Here, 200 µl of cells were transferred to 96-well plates and incubated overnight at 37°C, 5% CO_2_ to allow macrophages to adhere to the plate. Non-adherent cells were gently washed with RPMI medium, and 100 µl of fresh RPMI medium was added to each well. Furthermore, 25 µl of the polyherbal combination was added to the cells in each well and incubated for 48 h at 5% CO_2_ and 37°C. After incubation, 100 µl of neutral red solution (0.1% in 10 mM PBS) was added, and after 2 h of incubation, a free neutral red solution was removed by gently washing the cells with PBS. The macrophages were further lysed by adding 100 µl of neutral red detainer (ethanol and 0.1% acetic acid in a ratio of 1:1, v/v) and incubated at room temperature overnight, and optical density was recorded the next day at 540 nm (Shi and Fu, 2011). For negative control, RPMI medium was used in place of the combination, and the results were expressed as compared to the negative control.

### Immunoprotective Effect of the Polyherbal Combination

To determine the effect of the developed polyherbal combination on the immune system, BALB/c mice (25 ± 5 g) were used for the study. Animals were randomly divided into seven groups consisting of six animals per group. Groups I–III received normal saline for 14 days, and group I served as normal control. In addition to normal saline, Groups II and III received polyherbal combination at an oral dose of 260 and 520 mg/kg, respectively, for 14 days. Groups II and III served as a sham control of the polyherbal combination. Groups IV–VII received cyclophosphamide (80 mg/kg, i.p.) for 4 days of the experimental period (from days 8 to 11), followed by a washout and normalization period of cyclophosphamide for 3 days. In addition to cyclophosphamide, Group IV received normal saline throughout the experimental period and served as a toxic control; Group V and Group VI received polyherbal combination orally at a dose of 260 and 520 mg/kg, respectively; Group VII received levamisole hydrochloride at a dose of 10 mg/kg orally for 14 days and served as a positive control. The oral dose of the polyherbal combination was decided on the basis of the RSM result and administered as a suspension in 0.1% carboxymethylcellulose. At the end of the experiment, blood samples were collected from each animal from the retro-orbital vein and used for further analysis.

#### Determination of Fluorocytometry-Based Immunological Parameters

**FIGURE 1:**
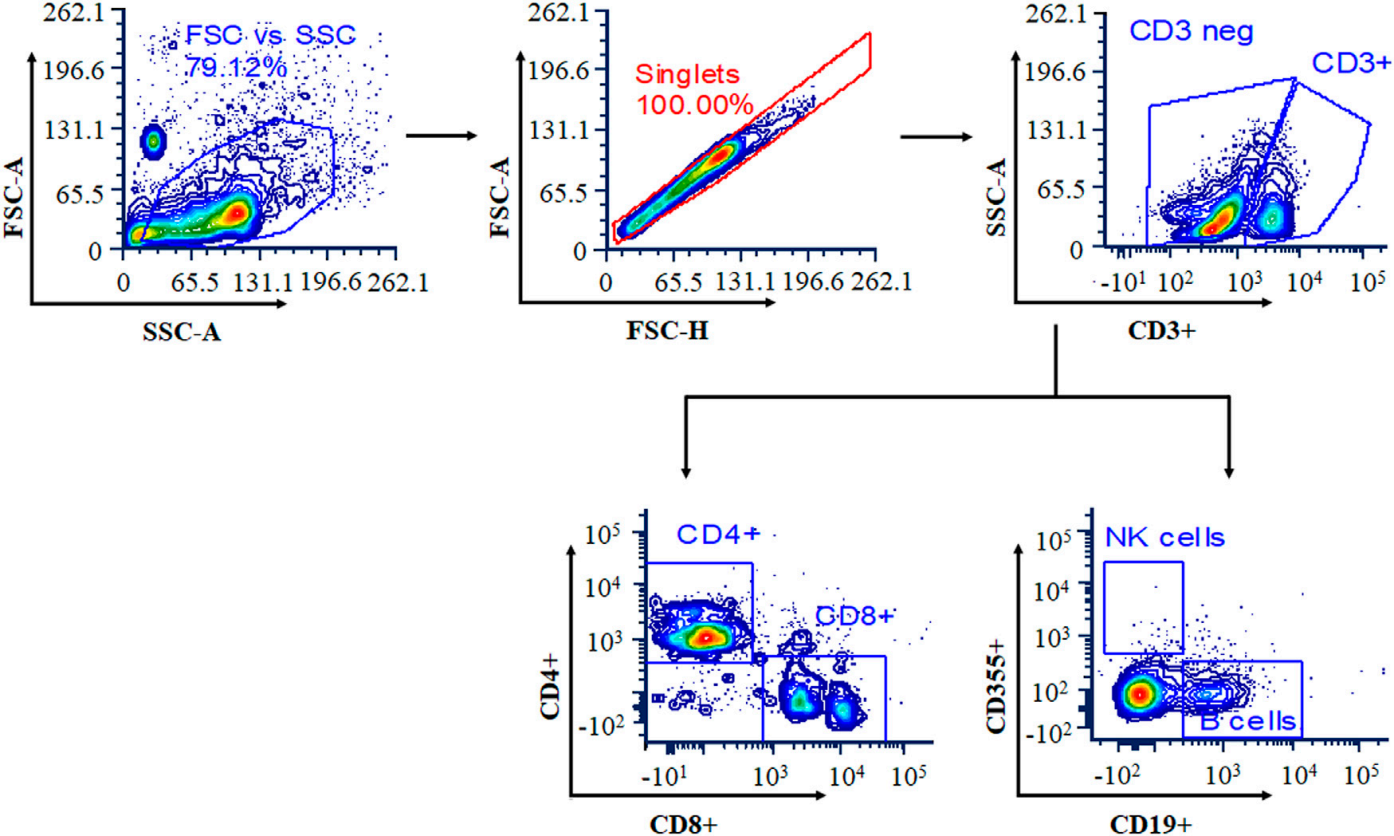
Gating strategy for selected populations of T cells, B cells, and natural killer (NK) cells. Cells were stained with a panel of cell-specific antibodies conjugated with different fluorochromes: CD3e-FITC, CD4-PE, CD8-APC, CD19-APC, and CD335-PE.

Blood samples were collected at the end of the 14-day study period in anticoagulant-containing collection tubes. The RBCs were lysed using ammonium-chloride-potassium (ACK) lysis buffer for 10 min at room temperature, and the reaction was stopped by adding RPMI medium. For flow cytometry analysis, cells were washed twice with cold FACS buffer and pelleted by centrifugation at 400 g for 5 min. Cells were resuspended in FACS buffer containing antibodies at 1:100 dilution (CD3e-FITC, CD4-PE-Cy7, CD8-APC-Cy7, CD19-APC, and CD335-PE) and incubated for 25 min at 4°C in the dark. After the incubation period, cells were washed twice with FACS buffer and resuspended in 200 µl of FACS buffer before being acquired on a BD LSR II flow cytometer (BD Bioscience, USA). Data were analyzed using FCS Express 7 Research Edition, and results were expressed in terms of percentage of CD4 cells, CD8 cells, natural killer (NK) cells, and B cells. The gating strategy for selected populations of T cells, B cells, and NK cells is shown in Figure 1.

#### Determination of Hematological Parameters

Blood samples from mice were collected in a tube containing ethylenediaminetetraacetic acid (EDTA), and white blood cell (WBC), RBC, hemoglobin (HGB), and platelet (PLT) counts were determined using a fully automated hematology analyzer (XP 100, Sysmex, Japan) (Anjum et al., 2017).

### Statistical Analysis

Data are shown as mean ± SEM, and the difference between groups was analyzed by two-way ANOVA followed by Bonferroni posttest using GraphPad Prism 5.00. A p-value <0.05 was considered statistically significant.

The ANOVA provision of Design Expert was used to establish statistical validation of the equation supplied by the software. Using BBD and the Design Expert 8.0.1.7 program (Stat-Ease Inc., USA), a total of 29 experimental combinations with three central points were created, and all of these combinations were evaluated for pinocytic activity and splenocyte proliferation assay. The models were assessed using *R* ^*2*^ values and statistically significant coefficients. The validation of the RSM results was carried out to determine the optimal combination compositions across the entire experimental region. ANOVA was used to justify the inadequacies of the models. After fitting the data to multiple models (linear, 2FI, quadratic, and cubic) and performing an ANOVA, it was found that the quadratic polynomial and 2FI models were the best fit for the splenocyte proliferation assay and pinocytic activity, respectively. The experimental values of the reactions were compared to the expected values quantitatively. MS-Excel was also used to create linear regression charts between the actual and predicted response values. The Box–Cox plot was used to describe the appropriate power transformation of the response data.

Principal component analysis (PCA) is a powerful statistical tool for data analysis and expression that can highlight the similarities and differences of sets. Raw data obtained from the UPLC-MS analysis were processed in the Progenies software (Waters, USA). Using this software, the maximum number of mass peaks can be captured and further identified by the library on the basis of their molecular weight. All detected metabolites are processed in the XLStat software to make a statistical difference between plants used to develop a polyherbal combination.

## Results

The plant materials used in the present study were authenticated according to the protocol of Indian Pharmacopoeia (Anonymous, 2010). The authenticated plant materials were extracted in three different solvents (aqueous, ethanol, and hydroethanol) by overnight maceration followed by reflux. We have evaluated aqueous, ethanolic, and hydroethanolic extracts from eight plants for their *in vitro* immunomodulatory activity. From eight plants, only aqueous extracts of *P. emblica*, *T. cordifolia*, *W. somnifera*, and *P. nigrum* were found to be the best immunomodulators on the basis of their *in vitro* immunomodulatory activity and extractive yield.

### Quantitative Estimation of Specific Markers

The aqueous extracts of four selected plants were chemically characterized by quantifying specific markers present in them. The linear regression calibration curves plotted between the peak area vs. concentration were linear for all standards, namely, gallic acid, berberine, piperine, and withaferin-A, with good linear relationships (*r* ^2^ = 0.99). Well-separated bands of gallic acid, berberine, piperine, and withaferin-A were visualized at *R*_f_ 0.44, 0.46, 0.69, and 0.35, respectively. HPTLC chromatograms of gallic acid, piperine, berberine, and withaferin-A in the optimized extract are shown in Supplementary Figures S1–S4. The percentages of gallic acid in *P. emblica*, piperine in *P. nigrum*, berberine in *T. cordifolia*, and withaferin-A in *W. somnifera* were found to be 5.01% ± 0.07%, 1.62% ± 0.01%, 1.04% ± 0.02%, and 2.35% ± 0.04%, respectively, of total weight (w/w).

### Response Surface Methodology-Based Optimization of Extract Ratio for the Development of the Polyherbal Combination

The aqueous extracts of these four plants were optimized in ratios by RSM to develop a polyherbal combination. A BBD with three levels was used for all four extracts of *P. emblica* (A), *P. nigrum* (B), *W. somnifera* (C), and *T. cordifolia* (D) to finalize their ratio for the development of a polyherbal combination. The range of variables, designs, and results obtained for splenocyte proliferation and pinocytic activity is presented in Table 1. The results were the average of three independent assays. The experimental results were modeled with a second-order quadratic and two-factor interaction (2FI) model to explain the dependence of *in vitro* immunomodulatory activity on different factors. The experimental data obtained from BBD to predict splenocyte proliferation and pinocytic activity are expressed by the following equation.

Splenocyte proliferation assay: 0.2292 + 0.04425 A − 0.009666667 B − 0.031833333 C − 0.0075 D − 0.05987 AB − 0.021125 AC − 0.0485 AD + 0.0395 BC − 0.024625 BD − 0.045375 CD − 0.080183333 A^2^ − 0.042433333 B^2^ + 0.005566667 C^2^ + 0.013066667 D^2^.

Pinocytic activity: 0.056965517 + 0.029958333 A + 0.018291667 B + 0.002208333 C + 0.014791667 D + 0.04925 AB − 0.041375 AC − 0.011 AD + 0.0335 BC − 0.016375 BD − 0.00675 CD.

ANOVA for optimizing the ratio of four extracts to develop a polyherbal combination showed that the regression model was significant and the lack of fit was insignificant for both the splenocyte proliferation assay and pinocytic activity (Table 2).

**TABLE 2:** ANOVA of the quadratic response surface model for splenocyte proliferation and response surface 2FI model for pinocytic activity in Box–Bhenken design experiments.

Parameters	For splenocyte proliferation	For pinocytic activity
**Regression**		
Sum of square	0.136	0.0402
Df	14	10
Mean squares	0.0097	0.004
F value	1.337	2.7203
P	0.297	0.0311
**Residual**		
Sum of square	0.102	0.0266
Df	14	18
Mean square	0.0073	0.00147
**Lack of fit test**		
Sum of square	0.0845	0.0126
Df	10	14
Mean squares	0.0084	0.0009
F value	1.901	0.2601
p-value	0.2805	0.973
Coefficient correlation (*r* ^2^)	0.572	0.6017
Coefficient of variation (CV %)	45.936	67.52
Adequate precision value	4.965 (>4)	7.221(>4)

**FIGURE 2:**
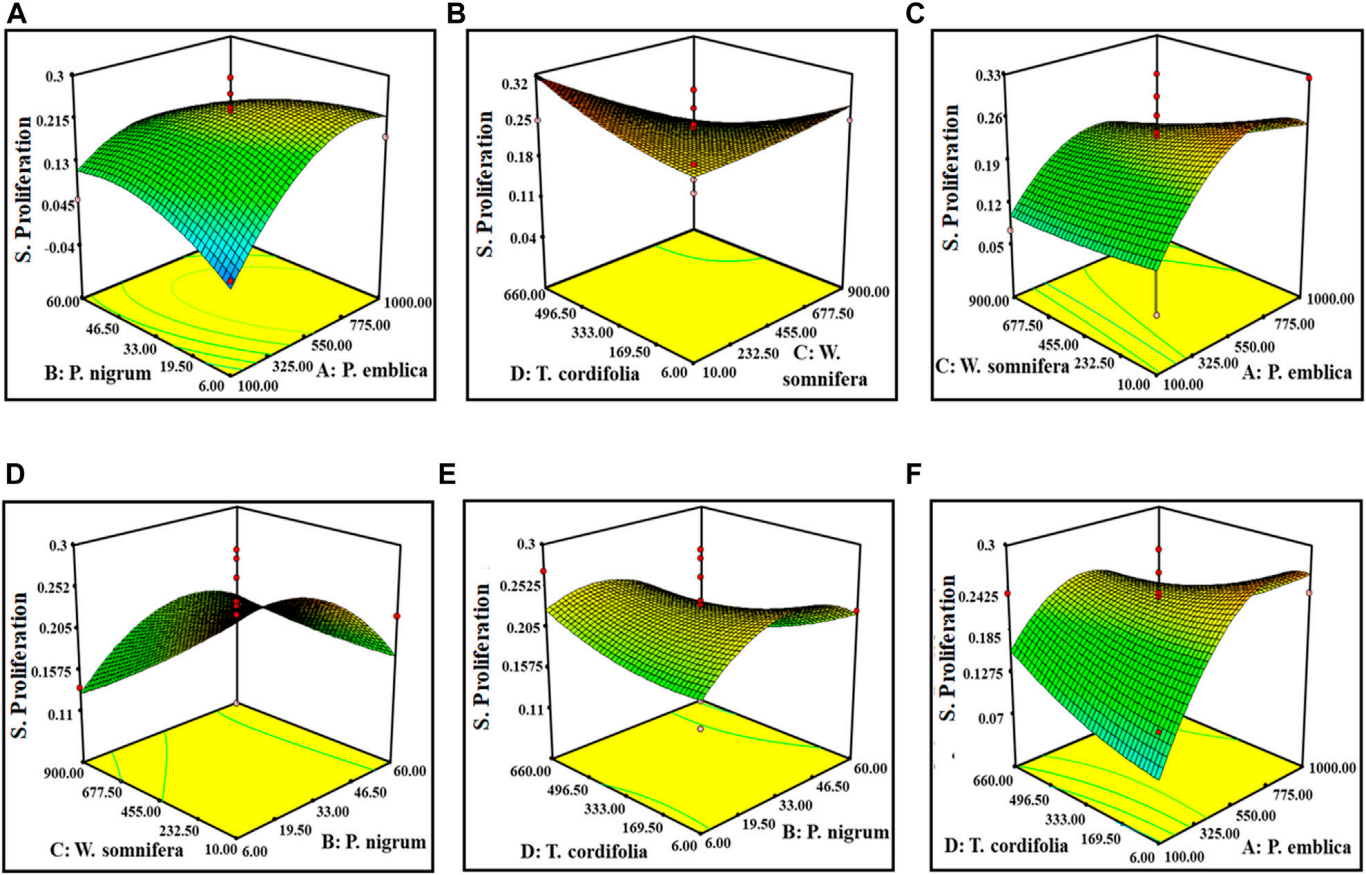
Response surface graph showing the interaction among four extract concentrations and splenocyte proliferation activity. Interaction between **(A)** *P. emblica* and *P. nigrum*, **(B)** *P. emblica* and *W. somnifera*, **(C)** *P. emblica* and *T. cordifolia*, **(D)** *P. nigrum* and *W. somnifera*, **(E)** *P. nigrum* and *T. cordifolia*, and **(F)** *W. somnifera* and *T. cordifolia*.

**FIGURE 3:**
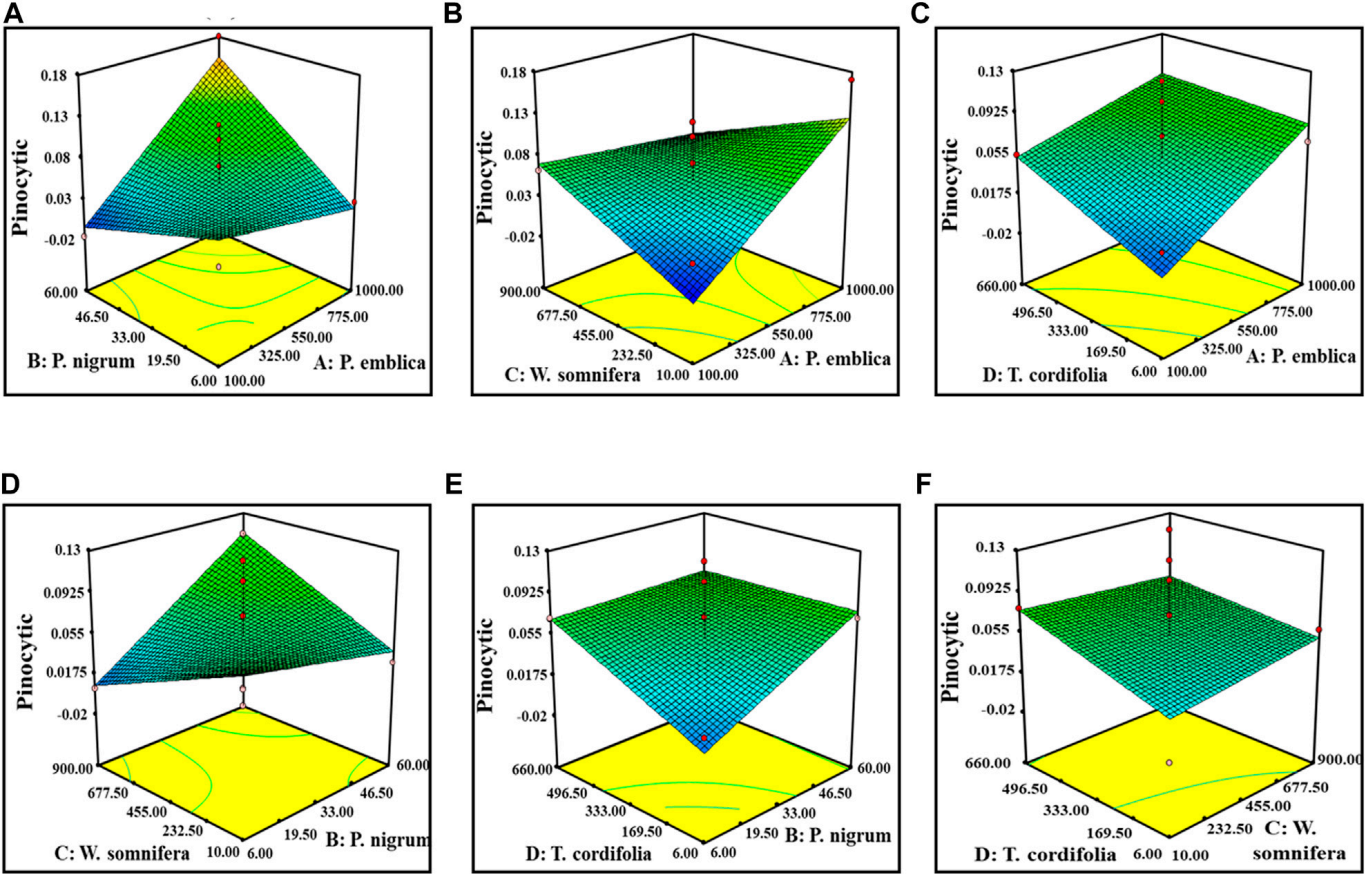
Response surface graph showing the interaction among four extract concentrations and pinocytic activity. Interaction between **(A)** *P. emblica* and *P. nigrum*, **(B)** *P. emblica* and *W. somnifera*, **(C)** *P. emblica* and *T. cordifolia*, **(D)** *P. nigrum* and *W. somnifera*, **(E)** *P. nigrum* and *T. cordifolia*, and **(F)** *W. somnifera* and *T. cordifolia*.

The Model F-value of 2.72 of pinocytic activity implies that the model is significant. There is only a 3.11% chance that a “Model F-value” this large could occur due to noise. The “Lack of Fit F-value” of 0.26 implies that the lack of fit is not significant relative to the pure error. A nonsignificant lack of fit is good for the model. The “Adeq Precision 7.22” indicating the excellent signal-to-noise ratio and ˃4 is desirable. This model is used to navigate the design space. However, for the splenocyte proliferation assay, the “Predicted *R* ^2^” of (−1.1524982) negative implies that the overall mean is a better predictor of the response than the current model. It showed Adeq precision of 4.96, indicating a good signal-to-noise ratio. Figures 2, 3 are the three-dimensional (3D) representation of the interacting effect of different plant extracts on the proliferation of splenocytes and pinocytic activity, respectively. A contour plot is a two-dimensional (2D) representation of the responses plotted against a combination of numerical factors and/or mixture components. It shows the relationship between the responses, the mixture components, and/or the statistical elements. Supplementary Figures S5–S6 represent the interactions among the various factors on splenocyte proliferation and pinocytic activity, respectively.

The optimal ratio of four different extracts for the maximum pinocytic activity and splenocyte proliferation assay was 49.76%, 1.35%, 5.41%, and 43.33%. for *P. emblica*, *P. nigrum*, *W. somnifera*, and *T. cordifolia*, respectively. The defined polyherbal combination predicted an improvement in splenocyte proliferation up to 32.7% and pinocytic activity by 11.8%. These optimized values of the ratio of four extracts were validated by *in vitro* immunomodulatory activity. The statistically developed polyherbal combination exhibited a 34% increase in splenocyte cell proliferation and 10.7% pinocytic activity, indicating a 90%–115% validation of the predicted model.

### Metabolomic Profiling of the Extract and the Developed Combination by Ultra-Performance Liquid Chromatography-Mass Spectrometry

UPLC-MS analysis files were processed through progenesis software to detect the maximum possible ions. Metlin, Massbank, and the chemical library were used to identify the ions and set tolerance levels below 10 ppm. In these four extracts and in combination, more than 5,000 ions were detected. These ions were from molecular ion peaks and/or may be from the fragmentation of molecular ion peaks. All of these ions were detected and processed through progenesis software and identified by a database based on their m/z values. From the whole mass range, the detected ions were statistically differentiated through the S-plot, and the major metabolites causing differentiation between one extract and another were screened. The main abundant metabolites of the extracts and the combination are summarized in Supplementary Table S1. Most of the metabolites found are also reported in these plant extracts. Some common and major metabolites of *P. emblica* are phyllanemblin (R_t_ 1.25), gallic acid (R_t_ 2.08), quercetin (R_t_ 6.02), caffeic acid (R_t_ 4.59), ellagic acid (R_t_ 4.13), and punigluconin (R_t_ 8.05). Similarly, digallic acid (R_t_ 2.92), 7-hydroxyflavanone (R_t_ 2.92), caffeic acid (R_t_ 4.59), schaftoside (R_t_ 4.56), syringin (R_t_ 4.59), jatrorrhizine (R_t_ 4.40), and sinapic acid (R_t_ 1.65) are found in *T. cordifolia*. Withaferin (R_t_ 5.25), withanolide (R_t_ 7.35), chlorogenic acid (R_t_ 4.16), and anaferine (R_t_ 6.30) were found in *W. somnifera*. Justicidin B (R_t_ 1.15), apiin (R_t_ 4.56), papaverine (R_t_ 4.78), piperazinamine (R_t_ 5.37), piperine (R_t_ 7.54), piperanine (R_t_ 9.78), and cubebin (R_t_ 7.69) are some metabolites found in *P. nigrum*. Some specific metabolites are found only in a single plant extract and a combination. The structure of major metabolites is summarized in Supplementary Figure S7. However, the results of UPLC-MS analysis were processed through PCA. As per the PCA plots (Supplementary Figure S8), all the aqueous extracts show different types of metabolites, and it can be seen in the distribution of the aqueous extract in a different quadrant. All of these compounds are not being detected in combination due to their abundance in the extract. Several metabolites are also found in all extracts and in combination. Gallic acid (R_t_ 2.08), quercetin (R_t_ 6.02), ellagic acid (R_t_ 4.13), caffeic acid (R_t_ 4.59), kaempferitrin (R_t_ 5.02), and *p*-coumaric acid (R_t_ 1.25) are some common and significant metabolites also found in all the extracts and in combination.

### Characterization of the Developed Polyherbal Combination

The optimized combination was characterized by determining their phenolic and flavonoid contents. Phenolic and flavonoid contents were expressed as gallic acid and rutin equivalent, respectively. The phenolic and flavonoid contents of the optimized combination were found to be 29.32% and 17.65% w/w of the polyherbal combination, respectively. The plants present in the polyherbal combination are the most abundant sources of phenolic and flavonoid metabolites, which may be responsible for their bioactivity (Mishra et al., 2013; Zhang et al., 2017; Tungmunnithum et al., 2018).

The developed polyherbal combination showed a potent DPPH radical scavenging potential, and IC_50_ was recorded as 82.54 ± 2.54 μg/ml and was comparable to a standard antioxidant compound, ascorbic acid (IC_50_ 55.37 ± 1.25). The IC_50_ of a compound is inversely proportional to its antioxidant activity, as it expresses the concentration of antioxidants required to decrease the concentration of DPPH by 50%. The presence of antioxidants in the herbal combination causes a reduction of Fe^3+^ to Fe^2+^ form. At 120 μg/ml, the polyherbal combination showed the maximum reducing power, which is equivalent to 80 μg/ml of ascorbic acid.

### *In Vitro* Immunomodulatory Activity of the Developed Polyherbal Combination

**FIGURE 4:**
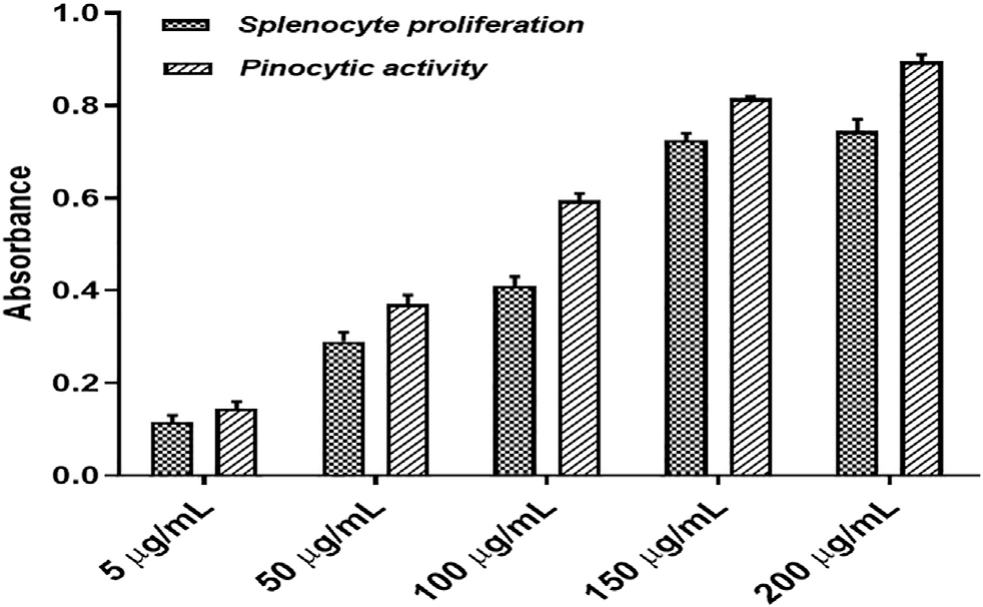
Splenocyte proliferation assay and pinocytic activity of the developed polyherbal combination.

The splenocytes isolated from the culture in RPMI medium for 72 h with or without polyherbal combination were counted for viability using the MTT assay. It was observed that splenocytes proliferate more when treated with the polyherbal combination compared to untreated cells. Here, five different concentrations (5–200 μg/ml) of the polyherbal combination were tested for the splenocyte proliferation assay, and it was found that there was a dose-dependent increase in cell proliferation up to the concentration of 150 μg/ml. In contrast, the concentration above 150 μg/ml showed no significant increment in the number of cells (Figure 4). However, all five concentrations of the combination showed dose-dependent pinocytic activity. Macrophages are the first-line defense against the antigen, and pinocytic activity is one of the distinguished methods to determine macrophage activation. We cultured mouse peritoneal macrophages in RPMI medium with or without supplementation of external stimuli and, after 48 h of incubation, used neutral red, which is readily taken up by macrophages to measure pinocytic activity. After the incubation with neutral red, the macrophages were lysed to measure the concentration of neutral red and determine the extent of pinocytic activity by macrophages. The enhancement of pinocytic activity by macrophages was expressed as the increase in neutral red concentration in cells treated with external stimuli as compared to untreated cells (Figure 4).

### Effect of the Polyherbal Combination on Immunological Parameters

#### Hematological Parameters

Without affecting HGB content and RBC count, a significant reduction in lymphocyte, WBC, and platelet counts was observed in cyclophosphamide-induced immunosuppressed mice. This change has been reversed and almost normalized upon administration of both doses of the polyherbal combination. Table 3 shows the hematological parameters in mice from all groups. Healthy mice treated with the polyherbal combination showed a slight increase in favorable immune cells such as platelets, lymphocytes, and WBCs.

**TABLE 3:** Effect of polyherbal combination on hematological parameters of mice after the end of treatment.

Hematological parameter	G-I	G-II	G-III	G-IV	G-V	G-VI	G-VII
HGB (g/dl)	11.22 ± 0.47	13.61 ± 0.77^###^	10.92 ± 0.64^ns^	11.82 ± 0.45^ns^	11.63 ± 0.98^ns^	12.21 ± 1.11^ns^	12.44 ± 0.54^ns^
Neutrophil (×10^3^/µl)	4.12 ± 0.08	5.87 ± 0.44^#^	4.14 ± 0.01^ns^	5.74 ± 0.22^#^	4.27 ± 0.05^ns^	4.88 ± 0.08^ns^	4.34 ± 0.07^ns^
Lymphocyte (×10^3^/µl)	7.23 ± 0.07	8.11 ± 0.14^ns^	9.33 ± 0.08^##^	4.25 ± 0.21^###^	5.45 ± 0.04^ns^	6.99 ± 0.05^***^	6.11 ± 0.14^*^
WBC (×10^3^/µl)	6.24 ± 0.09	6.91 ± 0.09^ns^	7.88 ± 0.07^#^	3.33 ± 0.14^###^	5.33 ± 0.11^**^	6.74 ± 0.45^***^	6.14 ± 0.11^***^
RBC (×10^6^/µl)	6.02 ± 0.12	6.47 ± 0.47^ns^	6.98 ± 0.45^ns^	6.06 ± 0.13^ns^	5.84 ± 0.02^ns^	6.94 ± 0.31^ns^	7.09 ± 0.23^ns^
PLT (×10^5^/µl)	7.45 ± 0.12	8.44 ± 1.11^ns^	12.36 ± 0.11^###^	5.93 ± 0.54^ns^	10.70 ± 0.45^**^	10.24 ± 0.47^***^	11.32 ± 0.45^***^

HGB, hemoglobin; PLT, platelet; RBC, red blood cell; WBC, white blood cell; G-I, Normal control; G-II, Combination low dose sham; G-III, Combination high dose sham; G-IV, Toxic control; G-V, Combination low. Dose; G-VI, Combination high dose; G-VII, Standard treated group. The data were indicated as mean ± SEM. Results were analyzed by two-way ANOVA, and the level of significant differences among different groups was determined by Bonferroni posttest using GraphPad Prism version 5.00 software. At 95% confidence interval, p < 0.05 was considered as statistically significant, ns p > 0.05, *p < 0.05, **p < 0.01, ***p < 0.001; hash “#” denoted that data were compared to normal control, and asterisk “*” denoted that data were compared to toxic control.

#### Immunomodulatory Parameters

**FIGURE 5:**
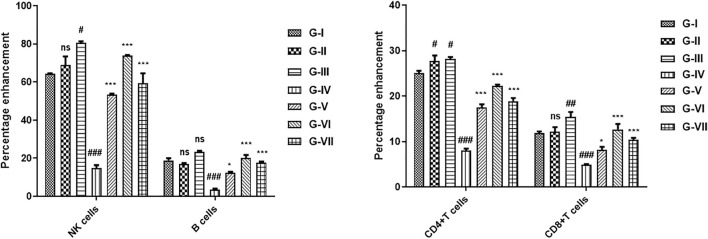
Effect of the developed polyherbal combination on cyclophosphamide-induced immunosuppressed mice. Data were obtained from FACS analysis, **(A)** natural killer (NK) cells and B cells, and **(B)** CD4 and CD8 cells. The data were indicated as mean ± SEM. Results were analyzed by two-way ANOVA, and the level of significant differences among different groups was determined by Bonferroni posttest using GraphPad Prism version 5.00 software. At 95% confidence interval, p < 0.05 was considered as statistically significant, ns p > 0.05, *p < 0.05, **p < 0.01, ***p < 0.001; hash “#” denoted that data were compared to normal control, and asterisk “*” denoted that data were compared to toxic control. G-I (Normal control), G-II (Sham low dose), G-III (Sham high dose), G-IV (Toxic control), G-V (Combination low dose), G-VI (Combination high dose), and G-VII (Standard).

The effect of the developed polyherbal combination on the immune system was measured by analyzing the percentage of increase or decrease in T cells, B cells, and NK cells. An increase in T-cell proliferation was observed in the cyclophosphamide-induced immunosuppressed mouse model when treated with the polyherbal combination (Figure 5). Cyclophosphamide treatment resulted in a significant reduction in CD4^+^ and CD8^+^ T cells, NK cells, and B cells. Two doses of the polyherbal combination (260 and 520 mg/kg) were administered orally to normal and immunosuppressed mice. Treatment with the developed polyherbal combination in cyclophosphamide-induced immunosuppressed mice resulted in a significant increase in the number of CD4^+^ and CD8^+^ T cells. Both doses, 260 and 520 mg/kg, increased CD4 cells by 18.25% and 22.56%, respectively, compared to 8.50% in the toxic control. A significant increase in the population of B cells was observed; similarly, a significant increase in NK cells was recorded in immunosuppressed mice treated with the polyherbal combination. The polyherbal combination elicited a proportional enhancement of the immune cell population, with the maximum response being at 520 mg/kg dose (Figure 5). In normal mice administered the polyherbal combination, a slight but not significant increase in the number of the immune cell population was observed, such as CD4^+^ T cells (2%), CD8^+^ T cells (3%), NK cells (9%), and B cells (3%).

## Discussion

In the Indian traditional system of medicine, more than 30 plants have been used, individually or in polyherbal combinations, for immune disorders. On the basis of the reported literature, we have chosen eight plant materials and screened them for immunomodulatory activity. The aqueous, ethanolic, and hydroethanolic extracts of these plants were screened for their splenocyte proliferation assay and pinocytic activity. Based on their extractive values and *in vitro* immunomodulatory activities, the aqueous extracts of four plants were selected for the development of a polyherbal immunomodulator, although hydroethanolic extractive values of some plants were found to be higher compared to aqueous and ethanolic extracts. In the present investigation, we considered only aqueous extracts because they are preferred over other preparations of traditional formulations and are considered much safer as compared to other extracts (Mensah et al., 2019). The aqueous extracts of these four plants underwent ratio optimization for the development of the polyherbal combination.

The application of RSM allowed the simultaneous determination of the main and interaction effects of different concentrations of all the extracts on *in vitro* pinocytic and splenocyte proliferation assay (Shahab et al., 2020). The data pertaining to various models (linear, 2FI, quadratic, and cubic) and their subsequent ANOVA revealed that splenocyte proliferation assay and pinocytic activity were the most suitably defined with the quadratic polynomial and 2FI models, respectively.

**FIGURE 6:**
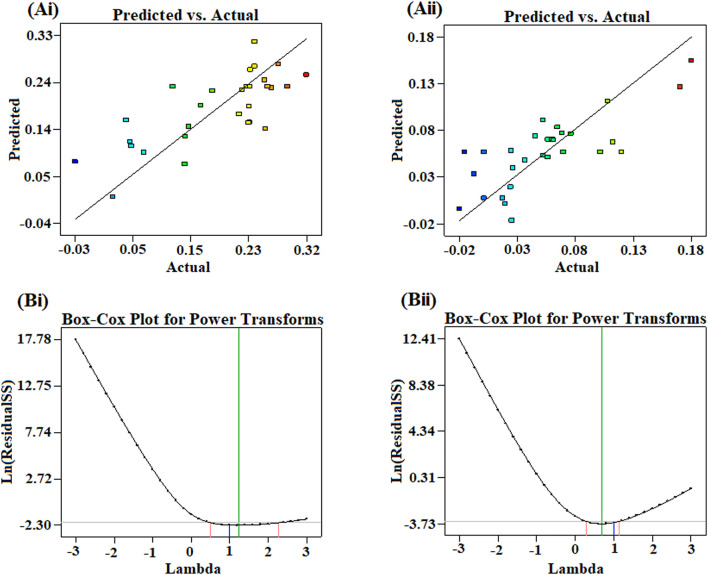
Plots showing the correlation of actual conversions and values predicted by the model for **(Ai)** splenocyte proliferation and **(Aii)** pinocytic activity. Box–Cox plot showing optimized lambda for the transformation of **(Bi)** splenocyte proliferation and **(Bii)** pinocytic activity.

The plot indicated that the residuals follow a normal distribution and approximately form a straight line (Figure 6). “Adequate Precision” is an index that measures the signal-to-noise ratio. A ratio of greater than 4 is desirable. In this study, the computed ratio of 4.96 and 7.21 for splenocyte proliferation assay and pinocytic activity, respectively, indicated an adequate signal. These results are indicative of maximum predictive responses with constant variance and quadratic model accuracy demonstrating a good reproducibility of the data. The optimum conditions were determined using RSM with a four-factor, two-level BBD. It was also estimated using regression equation for finding the best range of parameters achieving maximum splenocyte proliferation and pinocytic activity. To describe the suitable power transformation of response data, the Box–Cox plot was used (Vélez et al., 2015), since lambda (λ) is the most appropriate power transformation to apply the response data (Figure 6B).

From the point prediction tools, we have checked the effect of different doses of the extract. Upon increasing the dose of *P. emblica* up to 730 mg (100–730 mg), it increased the proliferation of the splenocytes and the pinocytic activity of macrophages, but after that point, the proliferation of splenocytes decreased but no significant change in the pinocytic activity. At an optimal level of *P. emblica* (750 mg), the increased dose of *P. nigrum* up to 22.2 mg was directly proportional to the *in vitro* immunomodulatory activity in both the assays. Furthermore, inverse proliferation was observed. At an optimal level of *P. emblica* (750 mg) and *P. nigrum* (20.59 mg), the concentration of *W. somnifera* was found inversely proportional to the *in vitro* immunomodulatory activity. At a low concentration of *T. cordifolia*, increased splenocyte proliferation and lower pinocytic activity were predicted when the levels of *P. emblica*, *P. nigrum*, and *W. somnifera* was at the optimal level. Individually, a dose of *P. nigrum* up to 41 mg increased splenocyte proliferation and decreased pinocytic activity, while, after this level, splenocyte proliferation was decreased, but no changes in pinocytic activity were measured. *W. somnifera* individually did not cause any change in pinocytic activity but was directly proportional to the proliferation of splenocytes. Similarly, *T. cordifolia* on a lower dose caused an increase in the proliferation of splenocytes and *vice versa* for pinocytic activity.

The combination has shown a better antioxidant potential compared to individual extracts. A lower value of IC_50_ (82.54 ± 2.54 μg/ml) indicates a higher antioxidant activity of the combination. The plant extracts used in the polyherbal combination contained a higher amount of phenolics and flavonoids and may be responsible for antioxidant activity (Liu et al., 2009). The antioxidant potential exhibited by the developed combination might be due to the presence of secondary metabolites from selected plants. In particular, *P. emblica* contains hydrolyzable tannins with low molecular weight having a very strong antioxidant action (Ghosal et al., 1996; Singh et al., 2015), whereas *W. somnifera* contains withanolides, naturally occurring steroidal lactones having strong antioxidant properties (Devkar et al., 2014; Zahiruddin et al., 2020). Similarly, a strong antioxidant potential was also reported for the aqueous extract of *T. cordifolia* (Premanath and Lakshmidevi, 2010) and *P. nigrum* (Gülçin, 2005). Our findings strongly suggest that these plant materials are promising sources of natural antioxidants.

The developed polyherbal combination showed not only immunomodulatory activity *in vitro* but also potential immunomodulatory activity against cyclophosphamide-induced immunosuppressed mice. Cyclophosphamide is known to cause a significant reduction in WBC and lymphocyte count (Huyan et al., 2011), and in this study, the effects of the polyherbal combination was tested on cyclophosphamide-induced immunosuppressed mice. The impact of the developed polyherbal combination was evaluated on HGB, neutrophils, lymphocytes, WBC, RBC, and platelet counts in cyclophosphamide-induced immunosuppressed mice. Lymphocyte, WBC, and platelet counts were significantly decreased upon cyclophosphamide treatment but reversed upon the treatment with the polyherbal combination. Even in healthy mice treated with the polyherbal combination, a slight increase in these cells was recorded.

WBCs are produced from the bone marrow, which is the most affected organ during immunosuppression therapy, as indicated by WBC and platelet counts (Vigila and Baskaran, 2008). Mice treated with cyclophosphamide showed a significant reduction in total platelet and WBC counts and recovered on treatment with both doses of combination. These results indicate that the herbal combination can modulate bone marrow activity, mainly suppression and stimulation, to counteract cyclophosphamide-induced myelosuppression. On the other hand, lymphocytes are a type of WBCs that are of fundamental significance. They regulate the specificity of the immune response to infectious microorganisms and other foreign bodies. Total lymphocyte counts were increased significantly in mice of all groups treated with the developed polyherbal combination compared to negative control mice. Our results also showed that there was no significant change in the RBC count in any group of mice. For a proper immune system, there should be harmony in RBC and WBC counts (El Bishlawy, 1999), and the developed polyherbal combination maintained the harmony. The results indicated that the developed polyherbal combination is highly efficient in augmenting the immune responses by enhancing the population of T cells and B cells but not NK cells. Since T cells play an important role in regulating the immune response, being responsible for cell-mediated immunity, in a balanced immune system, rapid T-cell proliferation following antigen stimulus is governed by subsequent differentiation of T cells into effectors cells (Luckheeram et al., 2012). Modifications of WBCs and differential counts are important signs of immune response and were supported by enhanced CD4 and CD8 cell counts.

The results of the developed polyherbal combination supported the traditional claims of plants used for an immunomodulatory activity. Previously, aqueous extracts of these four plant materials were tested for their immune enhancing potential. For example, the aqueous extract of *T. cordifolia* can modulate the immune system by increasing the count of WBC, and the aqueous extract is superior to the alcoholic extract (Manjrekar et al., 2000). Similarly, the root extract of *W. somnifera* also proved its T-cell enhancement, splenocyte cell proliferation, and stimulation of phagocytosis of macrophages (Davis and Kuttan, 2000). It has been also proven that the aqueous extract of *P. nigrum* causes splenocyte proliferation in a dose-dependent manner (Majdalawieh and Carr, 2010), and this report agrees with our *in vitro* results. The aqueous extract of these plant materials contains phenolic and flavonoids as the major group of constituents. These phenolic metabolites can promote nonspecific immune function, proliferate splenocytes, and enhance humoral immune responses and NK cell activity (Liu et al., 2012).

We have screened this developed polyherbal combination and the individual extracts for the identification of major metabolites. Of the identified metabolites, several metabolites were previously tested for immunomodulatory activity. Among different metabolites, cordifolioside A and tinocordiside present in the extract of *T. cordifolia* showed immunomodulatory activity (Sharma et al., 2012), and these compounds are also found in our developed polyherbal combination.

Gallic acid (R_t_ 2.08) and ellagic acid (R_t_ 4.13) have been reported to stimulate immune cells and can improve the immune cell population damaged by cyclophosphamide. Moreover, these compounds were both found in the extracts and also in the developed combination. Withaferin A (R_t_ 5.25) and withanolide (R_t_ 9.09) are the major metabolites found in the aqueous extract of *W. somnifera* and in the developed combination (Furmanowa et al., 2001). These two compounds activated phagocytosis and peritoneal macrophages, increased the secretion of lysosomal enzymes, and selectively enhanced the CD4 and CD8 counts (Bani et al., 2006). Asperuloside (R_t_ 9.47) increased the T-cell count, followed by increased secretion of interferon (IFN)-γ and tumor necrosis factor (TNF)-α, thereby enhancing the function of immune effector cells *via* induction of inflammatory cytokines (Chan et al., 2020). Asperuloside was found in *P. emblica* and *W. somnifera* extracts as well as in the developed polyherbal combination. Caffeic acid phenethyl ester (R_t_ 2.92) was found in all the extracts except *P. nigrum*. It increased T-lymphocyte production and enhanced the T cell-mediated immune response (Park et al., 2004). While caffeic acid (R_t_ 4.59) was present in all the extracts and also in the developed combination, it can increase NK cell activation and proliferate splenic T cells (Kilani-Jaziri et al., 2017).

Several formulations composed of these plant materials are available in the market claiming immunomodulatory activity. We have compared their activity with the results of the proposed combination on the basis of the reported literature. Gnanasekaran et al. (2015) reported that Chyawanprash could increase the total leukocyte in cyclophosphamide-induced immunosuppressed mice but not up to the normal level. Treatment with the developed combination of cyclophosphamide-induced immunosuppressed mice resulted in complete normalization of the total leukocyte count. Septilin, a well-known and widely used compound formulation in India, is composed of two powders and six plant extracts, in which *T. cordifolia* and *P. emblica* are the major ones. This formulation does cause the proliferation of splenocytes and increase in the lymphocyte count (Daswani and Yegnanarayan, 2002). But the level of significance is lower than the results obtained using our polyherbal combination. However, a real comparative analysis is ongoing in our laboratory. From these preliminary results, we can say that the developed polyherbal combination is better in terms of both *in vitro* and *in vivo* immune stimulating activity. The developed combination has been characterized by measuring the metabolomic content, thereby identifying the active constituents.

## Conclusions and Perspectives

The developed polyherbal combination containing *P. emblica*, *P. nigrum*, *W. somnifera*, and *T. cordifolia* exhibited potent immunomodulatory activity through stimulating pinocytosis and splenocyte proliferation. It also enhanced the subsets of various immune cells, mainly NK cells, B cells, CD4, and CD8 T cells, in cyclophosphamide-induced immunosuppressed mice. This immunomodulatory potential may be attributed to the presence of a group of metabolites in the polyherbal combination such as phenols, flavonoids, tannins, alkaloids, and glycosides exhibiting multiple mechanisms. The metabolomic profiling of the extracts and polyherbal combination through UPLC-MS revealed the presence of more than 180 metabolites, and PCA showed a wide array of metabolites, which are distributed in different quadrants. The polyherbal combination studied can be explored for its protective potential against various immunosuppressing clinical conditions and microbial attacks. Further research for its complete efficacy evaluation, development of its dosage form for its utilization in health care, and its in-depth metabolic characterization are in progress. It would be also interesting to check the pharmacokinetic profiling of the developed polyherbal combination with respect to specific marker constituents present in it.

## Data Availability

The raw data supporting the conclusions of this article will be made available by the authors, without undue reservation, to any qualified researcher.

## References

[B1] Alsuhaibani S. Khan M. A. (2017). Immune-Stimulatory and Therapeutic Activity of Tinospora Cordifolia: Double-Edged Sword against Salmonellosis. J. Immunol. Res. 2017, 1–9. 10.1155/2017/1787803 PMC5727750 29318160

[B2] Anjum V. Arora P. Ansari S. H. Najmi A. K. Ahmad S. (2017). Antithrombocytopenic and Immunomodulatory Potential of Metabolically Characterized Aqueous Extract of Carica Papaya Leaves. Pharm. Biol. 55, 2043–2056. 10.1080/13880209.2017.1346690 28836477 PMC6130488

[B3] Anonymous (2010). Indian Pharmacopoeia Commission, Ministry of Health and Family Welfare, 3. Ghaziabad: Government of India.

[B4] Bani S. Gautam M. Sheikh F. A. Khan B. Satti N. K. Suri K. A. (2006). Selective Th1 Up-Regulating Activity of Withania Somnifera Aqueous Extract in an Experimental System Using Flow Cytometry. J. Ethnopharmacol 107, 107–115. 10.1016/j.jep.2006.02.016 16603328

[B5] Bhalodia N. Nariya P. Shukla V. Acharya R. (2013). *In Vitro* antioxidant Activity of Hydro Alcoholic Extract from the Fruit Pulp of Cassia Fistula Linn. AYU 34, 209. 10.4103/0974-8520.119684 24250133 PMC3821253

[B6] Bui T. T. Piao C. H. Hyeon E. Fan Y. Van Nguyen T. Jung S. Y. (2019). The Protective Role of Piper Nigrum Fruit Extract in an Ovalbumin-Induced Allergic Rhinitis by Targeting of NFκBp65 and STAT3 Signalings. Biomed. Pharmacother. 109, 1915–1923. 10.1016/j.biopha.2018.11.073 30551446

[B7] Chan Y. Ng S. W. Xin Tan J. Z. Gupta G. Tambuwala M. M. Bakshi H. A. (2020). Emerging Therapeutic Potential of the Iridoid Molecule, Asperuloside: A Snapshot of its Underlying Molecular Mechanisms. Chemico-Biological Interactions 315, 108911. 10.1016/j.cbi.2019.108911 31786185

[B8] Chatterjee A. Chatterjee S. Biswas A. Bhattacharya S. Chattopadhyay S. Bandyopadhyay S. K. (2012). Gallic Acid Enriched Fraction ofPhyllanthus emblicaPotentiates Indomethacin-Induced Gastric Ulcer Healing via E-NOS-Dependent Pathway. Evid Based. Complement. Altern. Med. 2012, 1–13. 10.1155/2012/487380 PMC3433150 22966242

[B9] Daswani B. R. Yegnanarayan R. (2002). Immunomodulatory Activity of Septilin, a Polyherbal Preparation. Phytother. Res. 16, 162–165. 10.1002/ptr.996 11933120

[B10] Davis L. Kuttan G. (2000). Immunomodulatory Activity of Withania Somnifera. J. Ethnopharmacology 71 (1-2), 193–200. 10.1016/S0378-8741(99)00206-8 10904163

[B11] Devkar S. Jagtap S. Katyare S. Hegde M. (2014). Estimation of Antioxidant Potential of Individual Components Present in Complex Mixture ofWithania somnifera(Ashwagandha) Root Fraction by Thin-Layer Chromatography-2,2-Diphenyl-1-Picrylhdrazyl Method. J. Planar Chromatogr. - Mod. TLC 27, 157–161. 10.1556/JPC.27.2014.3.2

[B12] El Bishlawy I. M. (1999). Red Blood Cells, Hemoglobin and the Immune System. Med. Hypotheses 53, 345–346. 10.1054/mehy.1997.0778 10608271

[B13] Furmanowa M. Gajdzis-Kuls D. Ruszkowska J. Czarnocki Z. Obidoska G. Sadowska A. (2001). *In Vitro* Propagation of Withania Somnifera and Isolation of Withanolides with Immunosuppressive Activity. Planta Med. 67, 146–149. 10.1055/s-2001-11494 11301861

[B14] Ganju L. Karan D. Chanda S. Srivastava K. K. Sawhney R. C. Selvamurthy W. (2003). Immunomodulatory Effects of Agents of Plant Origin. Biomed. Pharmacother. 57 (7), 296–300. 10.1016/S0753-3322(03)00095-7 14499177

[B15] Ghosal S. Tripathi V. K. Chauhan S. (1996). ChemInform Abstract: Active Constituents of Emblica Officinalis. Part 1. The Chemistry and Antioxidative Effects of Two New Hydrolysable Tannins, Emblicanin A ( Ia) and B (Ib). ChemInform 27. 10.1002/chin.199647279

[B16] Gnanasekaran S. Sakthivel K. M. Chandrasekaran G. (2015). Immunostimulant and Chemoprotective Effect of Vivartana, a Polyherbal Formulation against Cyclophosphamide Induced Toxicity in Swiss Albino Mice. J. Exp. Ther. Oncol. 11, 51–61. 26259390

[B17] Gorgani L. Mohammadi M. Najafpour G. D. Nikzad M. (2017). Piperine-The Bioactive Compound of Black Pepper: From Isolation to Medicinal Formulations. Compr. Rev. Food Sci. Food Saf. 16, 124–140. 10.1111/1541-4337.12246 33371546

[B18] Gülçin İ. (2005). The Antioxidant and Radical Scavenging Activities of Black Pepper (Piper Nigrum) Seeds. Int. J. Food Sci. Nutr. 56, 491–499. 10.1080/09637480500450248 16503560

[B19] Huyan X. H. Lin Y. P. Gao T. Chen R. Y. Fan Y. M. (2011). Immunosuppressive Effect of Cyclophosphamide on white Blood Cells and Lymphocyte Subpopulations from Peripheral Blood of Balb/c Mice. Int. Immunopharmacol 11, 1293–1297. 10.1016/j.intimp.2011.04.011 21530682

[B20] Khan W. Parveen R. Chester K. Parveen S. Ahmad S. (2017). Hypoglycemic Potential of Aqueous Extract of Moringa Oleifera Leaf and *In Vivo* GC-MS Metabolomics. Front. Pharmacol. 8, 577. 10.3389/fphar.2017.00577 28955221 PMC5601078

[B21] Kilani-Jaziri S. Mokdad-Bzeouich I. Krifa M. Nasr N. Ghedira K. Chekir-Ghedira L. (2017). Immunomodulatory and Cellular Anti-Oxidant Activities of Caffeic, Ferulic, and P-Coumaric Phenolic Acids: A Structure-Activity Relationship Study. Drug Chem. Toxicol. 40, 416–424. 10.1080/01480545.2016.1252919 27855523

[B22] Kim O. K. Yoo S. A. Nam D.-E. Kim Y. Kim E. Jun W. (2014). Immunomodulatory Effects of Curcuma Longa L. Extract in LP-BM5 Murine Leukemia Viruses-Induced Murine Acquired Immune Deficiency Syndrome. J. Korean Soc. Food Sci. Nutr. 43, 1317–1324. 10.3746/jkfn.2014.43.9.1317

[B23] Kumar D. Arya V. Kaur R. Bhat Z. A. Gupta V. K. Kumar V. (2012). A Review of Immunomodulators in the Indian Traditional Health Care System. J. Microbiol. Immunol. Infect. 45, 165–184. 10.1016/j.jmii.2011.09.030 22154993

[B24] Liu S. Lin J. Wang C. Chen H. Yang D. (2009). Antioxidant Properties of Various Solvent Extracts from Lychee (Litchi Chinenesis Sonn.) Flowers. Food Chem. 114, 577–581. 10.1016/j.foodchem.2008.09.088

[B25] Liu X. Zhao M. Wu K. Chai X. Yu H. Tao Z. (2012). Immunomodulatory and Anticancer Activities of Phenolics from Emblica Fruit (Phyllanthus Emblica L.). Food Chem. 131, 685–690. 10.1016/j.foodchem.2011.09.063

[B26] Luckheeram R. V. Zhou R. Verma A. D. Xia B. (2012). CD4+T Cells: Differentiation and Functions. Clin. Develop. Immunol. 2012, 1–12. 10.1155/2012/925135 PMC3312336 22474485

[B27] Majdalawieh A. F. Carr R. I. (2010). *In Vitro* investigation of the Potential Immunomodulatory and Anti-cancer Activities of Black Pepper (Piper Nigrum) and Cardamom (Elettaria Cardamomum). J. Med. Food 13, 371–381. 10.1089/jmf.2009.1131 20210607

[B28] Manjrekar P. N. Jolly C. I. Narayanan S. (2000). Comparative Studies of the Immunomodulatory Activity of Tinospora Cordifolia and Tinospora Sinensis. Fitoterapia 71, 254–257. 10.1016/S0367-326X(99)00167-7 10844163

[B29] Mathai R. T. Tonse R. Kalekhan F. Colin M. D. Prabhu H. S. Rao S. (2015). “Amla in the Prevention of Aging,” in Foods and Dietary Supplements in the Prevention and Treatment of Disease in Older Adults. Waltham, MA: Academic Press, 29–35. 10.1016/B978-0-12-418680-4.00003-8

[B30] Mathew S. Kuttan G. (1999). Immunomodulatory and Antitumour Activities of Tinospora Cordifolia. Fitoterapia 70, 35–43. 10.1016/S0367-326X(98)00017-3

[B31] Mensah M. L. K. Komlaga G. Forkuo A. D. Firempong C. Anning A. K. Dickson R. A. (2019). “Toxicity and Safety Implications of Herbal Medicines Used in Africa,” in Herbal Medicine. 10.5772/intechopen.72437

[B32] Mishra A. Kumar S. Pandey A. K. (2013). Scientific Validation of the Medicinal Efficacy ofTinospora Cordifolia. Scientific World J. 2013, 1–8. 10.1155/2013/292934 PMC3885194 24453828

[B33] Mohammed Al-Ezzy R. Anee R. S. A. A. Ibrahim N. A. (2018). Assessments of Immunological Activity of Achillea Millefolium Methanolic Extract on Albino Male Mice. J. Pharm. Pharmacol. 6, 563–569. 10.17265/2328-2150/2018.06.002

[B34] Mondal S. Varma S. Bamola V. D. Naik S. N. Mirdha B. R. Padhi M. M. (2011). Double-Blinded Randomized Controlled Trial for Immunomodulatory Effects of Tulsi (Ocimum Sanctum Linn.) Leaf Extract on Healthy Volunteers. J. Ethnopharmacology 136, 452–456. 10.1016/j.jep.2011.05.012 21619917

[B35] More P. Pai K. (2011). Immunomodulatory Effects of Tinospora Cordifolia (Guduchi) on Macrophage Activation. Biol. Med. 3 (2), 134–140.

[B36] Nagoba B. Davane M. (2018). Natural Immunomodulators. J. Immunol. Microbiol. 2 (1), 2.

[B37] Neag M. A. Mocan A. Echeverría J. Pop R. M. Bocsan C. I. Crişan G. (2018). Berberine: Botanical Occurrence, Traditional Uses, Extraction Methods, and Relevance in Cardiovascular, Metabolic, Hepatic, and Renal Disorders. Front. Pharmacol. 9, 557. 10.3389/fphar.2018.00557 30186157 PMC6111450

[B38] Park J. H. Lee J. K. Kim H. S. Chung S. T. Eom J. H. Kim K. A. (2004). Immunomodulatory Effect of Caffeic Acid Phenethyl Ester in Balb/c Mice. Int. Immunopharmacology 4, 429–436. 10.1016/j.intimp.2004.01.013 15037220

[B39] Parveen B. Pillai K. K. Tamboli E. T. Ahmad S. (2015). Effect of Piperine on Pharmacokinetics of Sodium Valproate in Plasma Samples of Rats Using Gas Chromatography-Mass Spectrometry Method. J. Pharm. Bioall Sci. 7, 317. 10.4103/0975-7406.168036 PMC4678974 26681892

[B40] Premanath R. Lakshmidevi N. (2010). Studies on Anti-oxidant Activity of Tinospora Cordifolia (Miers.) Leaves Using *In Vitro* Models. J. Am. Sci. 6, 736–743.

[B41] Rehman J. Dillow J. M. Carter S. M. Chou J. Le B. Maisel A. S. (1999). Increased Production of Antigen-specific Immunoglobulins G and M Following *In Vivo* Treatment with the Medicinal Plants Echinacea Angustifolia and Hydrastis Canadensis. Immunol. Lett. 68, 391–395. 10.1016/S0165-2478(99)00085-1 10424448

[B42] Sarvanandaa L. Premarathna A. D. Karunarathnad S. C. (2018). Immunomodulatory Effect of Cardiospermum Halicacabum against Cancer. Biomed. J. Scientific Tech. Res. 10 (4), 7916–7919. 10.26717/bjstr.2018.10.001976

[B43] Shahab M. S. Rizwanullah M. Alshehri S. Imam S. S. (2020). Optimization to Development of Chitosan Decorated Polycaprolactone Nanoparticles for Improved Ocular Delivery of Dorzolamide: *In Vitro*, *Ex Vivo* and Toxicity Assessments. Int. J. Biol. Macromolecules 163, 2392–2404. 10.1016/j.ijbiomac.2020.09.185 32979440

[B44] Sharma U. Bala M. Kumar N. Singh B. Munshi R. K. Bhalerao S. (2012). Immunomodulatory Active Compounds from Tinospora Cordifolia. J. Ethnopharmacology 141, 918–926. 10.1016/j.jep.2012.03.027 22472109

[B45] Shi L. Fu Y. (2011). Isolation, Purification, and Immunomodulatory Activity *In Vitro* of Three Polysaccharides from Roots of Cudrania Tricuspidata. Acta Biochim. Biophys. Sinica 43, 418–424. 10.1093/abbs/gmr024 21467071

[B46] Singh M. Khan M. A. Khan M. S. Ansari S. H. Ahmad S. (2015). Quality Assessment and Evaluation of Iin-Vvitro Antioxidant Potential of Phyllanthus Emblica L. Indian J. Traditional Knowledge 14 (2), 265–272.

[B47] Singh N. Bhalla M. de Jager P. Gilca M. (2011). An Overview on Ashwagandha: A Rasayana (Rejuvenator) of Ayurveda. Afr. J. Trad. Compl. Alt. Med. 8, 208–213. 10.4314/ajtcam.v8i5S.9 PMC3252722 22754076

[B48] Tiwari S. Atluri V. S. R. Yndart Arias A. Jayant R. D. Kaushik A. Geiger J. (2018). Withaferin A Suppresses Beta Amyloid in APP Expressing Cells: Studies for Tat and Cocaine Associated Neurological Dysfunctions. Front. Aging Neurosci. 10, 291. 10.3389/fnagi.2018.00291 30356847 PMC6190869

[B49] Tungmunnithum D. Thongboonyou A. Pholboon A. Yangsabai A. (2018). Flavonoids and Other Phenolic Compounds from Medicinal Plants for Pharmaceutical and Medical Aspects: An Overview. Medicines (Basel) 5, 93. 10.3390/medicines5030093 PMC6165118 30149600

[B50] van der Nat J. M. Klerx J. P. van Dijk H. De Silva K. T. Labadie R. P. (1987). Immunomodulatory Activity of an Aqueous Extract of Azadirachta indica Stem Bark. J. Ethnopharmacol 19, 125–131. 10.1016/0378-8741(87)90036-5 3302545

[B51] Vélez J. I. Correa J. C. Marmolejo-Ramos F. (2015). A New Approach to the Box-Cox Transformation. Front. Appl. Math. Stat. 1, 12. 10.3389/fams.2015.00012

[B52] Vigila A. G. Baskaran X. (2008). Immunomodulatory Effect of Coconut Protein on Cyclophosphamide Induced Immune Suppressed Swiss Albino Mice. Ethnobotanical Leaflets.

[B53] Zahiruddin S. Basist P. Parveen A. Parveen R. Khan W. Gaurav (2020). Ashwagandha in Brain Disorders: A Review of Recent Developments. J. Ethnopharmacology 257, 112876. 10.1016/j.jep.2020.112876 32305638

[B54] Zahiruddin S. Khan W. Nehra R. Alam M. J. Mallick M. N. Parveen R. (2017). Pharmacokinetics and Comparative Metabolic Profiling of Iridoid Enriched Fraction of Picrorhiza Kurroa - an Ayurvedic Herb. J. Ethnopharmacology 197, 157–164. 10.1016/j.jep.2016.07.072 27469200

[B55] Zhang J. Miao D. Zhu W. F. Xu J. Liu W. Y. Kitdamrongtham W. (2017). Biological Activities of Phenolics from the Fruits of Phyllanthus Emblica L. (Euphorbiaceae). Chem. Biodivers 14, e1700404. 10.1002/cbdv.201700404 28960771

