# Supplementary material for: Metabolomic Profiling and Immunomodulatory Activity of a Polyherbal Combination in Cyclophosphamide-Induced Immunosuppressed Mice
Source: Front Pharmacol. 2022 Jan 3;12:647244. doi: 10.3389/fphar.2021.647244 (PMC8762268; doi:10.3389/fphar.2021.647244)
Supplement: Supplementary file 1 [file Table1.DOCX]

**Supplementary Table 1**. Metabolites of extract and developed polyherbal combination analysed by UPLC-MS. A: *Phyllanthus emblica*; B: *Tinospora cordifolia*; C: *Withania somnifera*; D: *Piper nigrum;* E: developed polyherbal combination.

| **S.N.** | **RT** | **m/z** | **Compound Name** | **Formula** | **A** | **B** | **C** | **D** | **E** |
| --- | --- | --- | --- | --- | --- | --- | --- | --- | --- |
| 1 | 1.15 | 342.1388 | 1D-myo-inositol 2-amino-2-deoxy-alpha-D-glucopyranoside | C_12_H_23_NO_10_ | + | - | - | - | - |
| 2 | 1.15 | 365.1030 | Justicidin B | C21H16O6 | - | + | - | + | - |
| 3 | 1.15 | 444.1679 | Dihydrofolate | C_19_H_21_N_7_O_6_ | + | - | - | - | - |
| 4 | 1.15 | 606.0851 | GDP-beta-L-galactose | C_16_H_25_N_5_O_16_P_2_ | + | - | - | - | - |
| 5 | 1.18 | 118.0862 | L-Norvaline | C_5_H_11_NO_2_ | + | - | - | + | - |
| 6 | 1.18 | 118.0862 | Trimethylglycine | C_5_H_11_NO_2_ | - | + | - | + | - |
| 7 | 1.18 | 194.0447 | 3-amino-4,7-dihydroxycoumarin | C_9_H_7_NO_4_ | + | - | - | + | + |
| 8 | 1.18 | 248.1130 | N-[(1S)-1-Carboxy-2-methylpropyl]-D-glutamic acid | C_10_H_17_NO_6_ | + | - | - | - | - |
| 9 | 1.18 | 248.1130 | Linamarin | C_10_H_17_NO_6_ | - | - | + | + | - |
| 10 | 1.18 | 325.1127 | Dehydrodiscretamine | C_19_H_18_NO_4_+ | - | + | - | - | + |
| 11 | 1.22 | 144.1019 | Stachydrine | C_7_H_13_NO_2_ | - | + | + | - |  |
| 12 | 1.25 | 174.1132 | N-acetyl-dl-leucine | C_8_H_15_NO_3_ | - | + | - | - | + |
| 13 | 1.25 | 165.1192 | p-coumaric acid | C_9_H_8_O_3_ | + | + | + | + | + |
| 14 | 1.25 | 199.1072 | Phyllanemblin A | C_9_H_10_O_5_ | + | - | - | - | + |
| 15 | 1.25 | 305.1339 | 2'-deoxymugineic acid | C_12_H_20_N_2_O_7_ | + | - | + | + | + |
| 16 | 1.30 | 124.0391 | Picolinic Acid | C_6_H_5_NO_2_ | - | - | - | + | + |
| 17 | 1.30 | 226.0715 | 2-amino-2-deoxyisochorismic acid | C_10_H_11_NO_5_ | - | - | + | - | + |
| 18 | 1.30 | 130.0859 | Cycloleucine | C_6_H_11_NO_2_ | - | + | - | - | - |
| 19 | 1.30 | 130.0859 | L-pipecolic acid | C_6_H_11_NO_2_ | - | - | - | + | + |
| 20 | 1.30 | 182.0809 | L-(-)-Tyrosine | C_9_H_11_NO_3_ | + | + | - | + | + |
| 21 | 1.30 | 188.0912 | N-Acetyl-6-oxo-L-norleucine | C_8_H_13_NO_4_ | + | + | - | - | - |
| 22 | 1.30 | 194.0809 | Gentioflavine | C_10_H_11_NO_3_ | + | - | - | + | + |
| 23 | 1.30 | 226.0715 | 4-amino-4-deoxychorismic acid | C_10_H_11_NO_5_ | + | - | - | + | + |
| 24 | 1.34 | 315.0729 | Geranyl Pyrophosphate | C_10_H_20_O_7_P_2_ | + | - | - | + | - |
| 25 | 1.40 | 140.0349 | 6-Hydroxynicotinate | C_6_H_5_NO_3_ | - | - | - | + | - |
| 26 | 1.53 | 142.0492 | Isopellertierine | C_8_H_15_NO | + | + | - | - | + |
| 27 | 1.65 | 225.2502 | Sinapic acid | C_11_H_12_O_5_ | - | + | - | - | + |
| 28 | 1.65 | 438.8941 | - |  | + | - | - | - | - |
| 29 | 1.92 | 140.0341 | 6-Hydroxynicotinate | C_6_H_5_NO_3_ | - | - | - | + | + |
| 30 | 1.96 | 177.0542 | Ascorbic acid | C_6_H_8_O_6_ | + | - | - | - | + |
| 31 | 1.96 | 177.0542 | Hymecromone | C_10_H_8_O_3_ | - | + | - | - | - |
| 32 | 2.02 | 170.9957 | 3-sulfolactic acid | C_3_H_6_O_6_S | + | - | - | - | - |
| 33 | 2.08 | 171.0289 | Gallic acid | C_7_H_6_O_5_ | + | + | + | + | + |
| 34 | 2.08 | 383.0077 | Quercetin 3-O-sulfate | C_15_H_10_O_10_S | + |  | - | + | + |
| 35 | 2.58 | 220.1174 | Pantothenic acid | C_9_H_17_NO_5_ | - | + | - | - | - |
| 36 | 2.89 | 189.0867 | Aceglutamide | C_7_H_12_N_2_O_4_ | - | + | - | - | - |
| 37 | 2.89 | 291.1218 | 5-O-Methylvisamminol | C_16_H_18_O_5_ | - | - | - | + | - |
| 38 | 2.89 | 299.1267 | Enterolactone | C_18_H_18_O_4_ | - | + | - | - | + |
| 39 | 2.92 | 199.0752 | Capillarin | C_13_H_10_O_2_ | - | + | - | - | - |
| 40 | 2.92 | 165.0391 | 2,4-Dihydroxypteridine | C_6_H_4_N_4_O_2_ | - | - | - | + | + |
| 41 | 2.92 | 231.1007 | Demethylsuberosin | C_14_H_14_O_3_ | - | + | - | - | + |
| 42 | 2.92 | 241.0851 | 7-Hydroxyflavanone | C_15_H_12_O_3_ | - | + | - | + | + |
| 43 | 2.92 | 243.1006 | 7,4'-dihydroxyflavan | C_15_H_14_O_3_ | - | + | - | - | + |
| 44 | 2.92 | 257.1161 | Pterostilbene | C_16_H_16_O_3_ | - | + | - | - | + |
| 45 | 2.92 | 259.0957 | Hibiscoquinone A | C_15_H_14_O_4_ | - | - | - | + | + |
| 46 | 2.92 | 273.1113 | Vestitol | C_16_H_16_O_4_ | - | + | - | - | - |
| 47 | 2.92 | 285.1113 | Caffeic acid phenethyl ester | C_17_H_16_O_4_ | + | + | - | + | + |
| 48 | 2.92 | 289.1073 | Shikonin | C_16_H_16_O_5_ | - | + | - | - | + |
| 49 | 2.92 | 317.1378 | Sorgolactone | C_18_H_20_O_5_ | - | - | + | - | - |
| 50 | 2.92 | 323.0394 | Digallic acid | C_14_H_10_O_9_ | + | - | - | + | + |
| 51 | 2.92 | 334.1639 | Seneciphylline | C_18_H_23_NO_5_ | - | + | - | - | + |
| 52 | 3.20 | 298.1428 | Apoglaziovine | C_18_H_19_NO_3_ | + | - | - | - | - |
| 53 | 3.20 | 298.1428 | Glaziovine | C_18_H_19_NO_3_ | - | - | - | + | - |
| 54 | 3.35 | 315.0712 | (E)-Dantrolene | C_14_H_10_N_4_O_5_ | + | - | - | + | + |
| 55 | 3.35 | 372.1809 | Isoandrocymbine | C_21_H_25_NO_5_ | - | - | - | + | - |
| 56 | 3.66 | 249.1481 | Micheliolide | C_15_H_20_O_3_ | + | - | + | - | - |
| 57 | 3.94 | 370.0542 | Acetosulfone | C_14_H_15_N_3_O_5_S_2_ | - | + | - | - | - |
| 58 | 3.94 | 393.1547 | Vernodalol | C_20_H_24_O_8_ | - | + | - | - | - |
| 59 | 4.01 | 314.1151 | (-)-Salinosporamide A | C_15_H_20_ClNO_4_ | - | + | - | - | + |
| 60 | 4.09 | 371.1534 | Amylose | C_14_H_26_O_11_ | + | - | - | - | - |
| 61 | 4.13 | 303.0132 | Ellagic acid | C_14_H_6_O_8_ | + | + | + | + | + |
| 62 | 4.13 | 595.1649 | Isoorientin 2''-O-rhamnoside | C_27_H_30_O_15_ | + | - | - | - | + |
| 63 | 4.13 | 595.1649 | 2''-O-(beta-D-glucosyl) isovitexin | C_27_H_30_O_15_ | - | - | - | + | + |
| 64 | 4.13 | 595.1649 | Saponarin | C_27_H_30_O_15_ | - | + | - | - | - |
| 65 | 4.16 | 355.4123 | Chlorogenic acid | C_16_H_18_O_9_ | - | - | + | - | - |
| 66 | 4.16 | 386.1592 | Polycarpine | C_21_H_23_NO_6_ | - | - | - | + | + |
| 67 | 4.20 | 553.3699 | N-Acetyl-L-leucyl-L-isoleucylglycyl-L-valyl-N-methyl-L-prolinamide | C_27_H_48_N_6_O_6_ | - | - | - | + | + |
| 68 | 4.40 | 339.43 | Jatrorrhizine | C_20_H_20_NO_4_ | - | + | - | - | - |
| 69 | 4.40 | 370.1637 | Allocryptopine | C_21_H_23_NO_5_ | - | - | - | + | - |
| 71 | 4.44 | 757.0861 | P(1),P(2)-bis(5'-adenosyl) triphosphate | C_20_H_27_N_10_O_16_P_3_ | + | - | - | - | - |
| 72 | 4.47 | 261.0388 | Bellidin | C_13_H_8_O_6_ | + | - | - | - | + |
| 73 | 4.56 | 497.3111 | 20,26-dihydroxyecdysone | C_27_H_44_O_8_ | - | + | - | - | - |
| 74 | 4.56 | 565.1570 | Apiin | C_26_H_28_O_14_ | - | - | - | + | + |
| 75 | 4.56 | 565.1570 | Schaftoside | C_26_H_28_O_14_ | - | + | - | - | + |
| 76 | 4.59 | 165.0693 | 1,3,5-Heptatriynyl-benzene | C_13_H_8_ | - | - | - | + | - |
| 77 | 4.59 | 181.0530 | Caffeic acid | C_9_H_8_O_4_ | + | + | + | + | + |
| 78 | 4.59 | 223.0732 | L-Cystathionine | C_7_H_14_N_2_O_4_S | + | - | + | + | - |
| 79 | 4.59 | 261.1131 | 8-deoxylactucin | C_15_H_16_O_4_ | - | + | - | - | - |
| 80 | 4.59 | 297.1126 | Calophyllin B | C_18_H_16_O_4_ | + | + | + | - | + |
| 81 | 4.59 | 297.1126 | Dasytrichone | C_18_H_16_O_4_ | - | - | - | + | - |
| 82 | 4.59 | 337.1066 | Berberine | C_20_H_18_NO_4_+ | - | + | - | - | - |
| 83 | 4.59 | 342.1703 | (-)-Isocorypalmine | C_20_H_23_NO_4_ | - | - | - | + | + |
| 84 | 4.59 | 345.1341 | Tembetarine | C_20_H_26_NO_4_+ | - | + | - | - | - |
| 85 | 4.59 | 345.1341 | 4',5,6,7-Tetramethoxyflavanone | C_19_H_20_O_6_ | - | - | - | + | - |
| 86 | 4.59 | 373.1287 | Syringin | C_17_H_24_O_9_ | - | + | - | - | + |
| 87 | 4.59 | 390.1554 | Senaetnine | C_20_H_23_NO_7_ | - | + | - | - | + |
| 88 | 4.63 | 140.0475 | 2-methylaminoethylphosphonic acid | C_3_H_10_NO_3_P | - | + | - | - | - |
| 89 | 4.63 | 199.0609 | Danshensu | C_9_H_10_O_5_ | - | + | - | - | - |
| 90 | 4.78 | 338.1382 | Isoficine | C_20_H_19_NO_4_ | - | + | - | - | + |
| 91 | 4.78 | 340.1533 | Papaverine | C_20_H_21_NO_4_ | - | - | - | + | - |
| 92 | 4.78 | 295.0968 | Dehydrocycloguanandin | C_18_H_14_O_4_ | + | - | - | - | + |
| 93 | 4.78 | 340.1533 | Nantenine | C_20_H_21_NO_4_ | - | + | - | - | - |
| 94 | 4.82 | 449.1068 | Quercitrin | C_21_H_20_O_11_ | + | - | - | + | + |
| 95 | 4.82 | 330.1685 | S-(+)-Reticuline | C_19_H_23_NO_4_ | - | + | - | - | + |
| 96 | 4.82 | 330.1685 | Sinomenine | C_19_H_23_NO_4_ | - | - | - | + | - |
| 97 | 4.87 | 785.0796 | Pedunculagin | C_34_H_24_O_22_ | + | - | - | - | - |
| 98 | 4.90 | 315.0877 | 4',6-Dihydroxy-5,7-dimethoxyflavone | C_17_H_14_O_6_ | - | - | - | - | - |
| 99 | 4.90 | 315.0877 | Irisolidone | C_17_H_14_O_6_ | - | - | - | + | - |
| 100 | 4.90 | 503.2065 | 3-methoxy-2,3-dihydrowithaferin | C_29_H_43_O_7_ | + | - | + | - | - |
| 101 | 5.02 | 579.1733 | Kaempferitrin | C_27_H_30_O_14_ | + | + | + | + | + |
| 102 | 5.06 | 497.3108 | 20,26-dihydroxyecdysone | C_27_H_44_O_8_ | - | + | - | - | - |
| 103 | 5.09 | 199.0600 | Vanillyl mandelic acid | C_9_H_10_O_5_ | - | - | - | + | - |
| 104 | 5.13 | 181.0506 | 3-(4-Hydroxyphenyl)Pyruvate | C_9_H_8_O_4_ | - | - | - | + | - |
| 105 | 5.18 | 125.0602 | 3-Hydroxybenzyl Alcohol | C_7_H_8_O_2_ | - | - | - | + | - |
| 106 | 5.25 | 433.1129 | Apigetrin | C_21_H_20_O_10_ | + | - | - |  | + |
| 107 | 5.25 | 433.1129 | Isovitexin | C21H20O10 | - | - | - | + | - |
| 108 | 5.25 | 471.7377 | Withaferin A | C_28_H_38_O_6_ | - | - | + | - | + |
| 109 | 5.33 | 591.2442 | - | C_31_H_34_N_4_O_8_ | - | - | - | - | - |
| 110 | 5.37 | 371.2215 | 4-(Diphenylmethyl)-N-((6-methyl-2-pyridinyl)methylene)-1-piperazinamine | C_24_H_26_N_4_ | - | - | - | + | - |
| 111 | 5.37 | 481.3155 | 26-Hydroxyecdysone | C_27_H_44_O_7_ | - | + | - | - | + |
| 112 | 5.37 | 481.3155 | Ajugasterone C | C_27_H_44_O_7_ | - | - | + | - | + |
| 113 | 5.64 | 397.6284 | Tinocordiside | C_21_H_32_O_7_ | - | + | - | - | + |
| 114 | 5.64 | 357.1335 | Kievitone | C_20_H_20_O_6_ | + | - | + | - | + |
| 115 | 5.64 | 375.1435 | Plaunol D | C_20_H_22_O_7_ | + | + | - | - | + |
| 116 | 5.68 | 356.1846 | (S)-(-)-tetrahydropalmatine | C_21_H_25_NO_4_ | - | - | - | - | - |
| 117 | 5.90 | 219.1013 | 6-Hydroxytremetone | C_13_H_14_O_3_ | - | - | + | - | - |
| 118 | 5.90 | 495.3303 | Makisterone A | C_28_H_46_O_7_ | - | + | - | - | + |
| 119 | 6.02 | 303.0501 | Quercetin | C_15_H_10_O_7_ | + | + | + | + | + |
| 120 | 6.02 | 303.0501 | 6-Hydroxyluteolin | C_15_H_10_O_7_ | + | - | - | + | + |
| 121 | 6.14 | 407.1342 | Astringin | C_20_H_22_O_9_ | + | + | - | - | - |
| 122 | 6.30 | 225.4267 | Anaferine | C_13_H_24_N_2_O | - | - | + | - | - |
| 123 | 6.30 | 311.1277 | 7-Hydroxy-3-(4-methoxyphenyl)-4-propylcoumarin | C_19_H_18_O_4_ | + | - | - | - | + |
| 124 | 6.30 | 359.1503 | Isocolumbin | C_20_H_22_O_6_ | - | + | - | - | - |
| 125 | 6.30 | 359.1503 | (+)-Pinoresinol | C_20_H_22_O_6_ | + | - | - | - | - |
| 126 | 6.33 | 173.0955 | Capillone | C_12_H_12_O | + | - | - | - | - |
| 127 | 6.33 | 233.1531 | Frullanolide | C_15_H_20_O_2_ | - | + | - | - | - |
| 128 | 6.61 | 316.1544 | Norreticuline | C_18_H_21_NO_4_ | - | + | - | + | + |
| 129 | 6.64 | 405.1557 | Mallotophenone | C_21_H_24_O_8_ | - | + | - | - | - |
| 130 | 6.73 | 338.1386 | Annuloline | C_20_H_19_NO_4_ | + | - | + | - | + |
| 131 | 6.73 | 338.1386 | Ficine | C_20_H_19_NO_4_ | - | + | - | - | + |
| 132 | 6.95 | 219.1018 | 6-Hydroxytremetone | C_13_H_14_O_3_ | + | - | - | + | - |
| 133 | 7.04 | 287.1965 | N-Acetylleucylleucine | C_14_H_26_N_2_O_4_ | - | + | - | + | + |
| 134 | 7.04 | 327.1239 | Methylophiopogonone B | C_19_H_18_O_5_ | - | + | - | + | + |
| 135 | 7.04 | 373.1284 | Sinensetin | C_20_H_20_O_7_ | + | - | - | - | - |
| 136 | 7.04 | 391.1390 | Populin | C_20_H_22_O_8_ | - | - | - | - | - |
| 137 | 7.26 | 314.1383 | Laurolistine | C_18_H_19_NO_4_ | - | + | - | - | + |
| 138 | 7.26 | 314.1383 | Moupinamide | C_18_H_19_NO_4_ | - | - | - | + | + |
| 139 | 7.26 | 336.1249 | 12*β*,15a-Dihydroxy methyltestosterone | C_14_H_23_Cl_2_N_3_O_2_ | - | - | + | - | - |
| 140 | 7.26 | 177.0555 | Herniarin | C_10_H_8_O_3_ | - | - | - | + | + |
| 141 | 7.35 | 455.1806 | Withanolide B | C_28_H_38_O_5_ | - | - | + | - | + |
| 142 | 7.51 | 419.0949 | 5'-Butyrylphosphoinosine | C_14_H_19_N_4_O_9_P | - | - | + | - | + |
| 143 | 7.54 | 505.6160 | Cordifolioside A | C_22_H_32_O_13_ | - | + | - | - | + |
| 144 | 7.54 | 247.1089 | N-Acetyl-D Musizin -Tryptophan | C_22_H_32_O_13_ | + | - | - | + | - |
| 145 | 7.54 | 286.3930 | Piperine | C_17_H_19_NO_3_ | - | - | - | + | + |
| 146 | 7.54 | 311.1278 | 7-Hydroxy-3-(4-methoxyphenyl)-4-propylcoumarin | C_19_H_18_O_4_ | + | - | - | + | + |
| 147 | 7.54 | 311.1278 | Porphyrin | C_20_H_14_N_4_ | - | - | - | - | + |
| 148 | 7.66 | 347.1495 | (+)-Gibberellic acid | C_19_H_22_O_6_ | + | - | - | - | + |
| 149 | 7.66 | 347.1495 | Hallactone A | C_19_H_22_O_6_ | - | - | + | - | - |
| 150 | 7.66 | 347.1495 | Cynaropicrin | C_19_H_22_O_6_ | - | + | - | - | + |
| 151 | 7.69 | 275.1119 | Diprogulic acid | C_12_H_18_O_7_ | + | + | + |  | + |
| 152 | 7.69 | 303.1083 | Terizidone | C_14_H_14_N_4_O_4_ | - | - | + | - | + |
| 153 | 7.69 | 357.1335 | Leachianone G | C_20_H_20_O_6_ | - | + | - | - | - |
| 154 | 7.69 | 352.1536 | Ochotensine | C_21_H_21_NO_4_ | + | - | - | - | + |
| 155 | 7.69 | 352.1536 | Benfluorex | C_19_H_20_F_3_NO_2_ | - | - | - | + | + |
| 156 | 7.69 | 357.1335 | (-)-Cubebin | C_20_H_20_O_6_ | - | - | - | + | + |
| 157 | 7.69 | 375.1431 | Budlein A | C_20_H_22_O_7_ | + | - | - | - | + |
| 158 | 6.80 | 233.1529 | (+)-Costunolide | C_15_H_20_O_2_ | - | + | - | - | + |
| 159 | 8.01 | 329.1377 | Decursin | C_19_H_20_O_5_ | - | + | + | + | + |
| 160 | 8.04 | 327.1230 | Methylophiopogonone B | C_19_H_18_O_5_ | + | + | + | - | - |
| 161 | 8.04 | 803.556 | Punigluconin | C_20_H_22_O_6_ | + | - | - | - | - |
| 162 | 8.04 | 359.1485 | Dihydrocubebin | C_20_H_22_O_6_ | - | - | - | + | + |
| 163 | 8.04 | 359.1485 | Isocolumbin | C_20_H_22_O_6_ | - | + | - | - | + |
| 164 | 8.04 | 399.1427 | Anthricin | C_22_H_22_O_7_ | + | - | - | - | + |
| 165 | 8.13 | 489.1878 | Viscosa lactone B | C_28_H_40_O_7_ | - | - | + | - | + |
| 166 | 8.16 | 405.1992 | Tetraxetan | C_16_H_28_N_4_O_8_ | - | - | + | - | + |
| 167 | 8.35 | 607.2123 | Ambenonium chloride | C_28_H_42_Cl_4_N_4_O_2_ | - | - | + | - | - |
| 168 | 8.38 | 308.2208 | Dihydrocapsaicin | C_18_H_29_NO_3_ | - | - | - | + | - |
| 169 | 8.38 | 646.2720 | Prednimustine | C_35_H_45_Cl_2_NO_6_ | - | - | - | - | - |
| 170 | 8.59 | 217.1581 | Atractylone | C_15_H_20_O | + | - | - | - | + |
| 171 | 8.69 | 279.1370 | Amprolium | C_14_H_19_ClN_4_ | - | - | - | + | + |
| 172 | 8.75 | 359.1496 | Miroestrol | C_20_H_22_O_6_ | + | + | + | - | - |
| 173 | 8.78 | 633.8421 | Sitoindoside IX | C_34_H_48_O_11_ | - | - | + | - | + |
| 174 | 9.09 | 489.2306 | Withanolide | C_30_H_32_ClN_2_O_2_ | - | - | + | - | + |
| 175 | 9.16 | 383.1343 | Farnesyl Diphosphate | C_15_H_28_O_7_P_2_ | - | - | - | - | + |
| 176 | 9.28 | 692.2959 | Maitansine | C_34_H_46_ClN_3_O_10_ | + | - | + | - | + |
| 177 | 9.31 | 408.1474 | Pagoclone | C_23_H_22_ClN_3_O_2_ | + | + | - | - | - |
| 178 | 9.37 | 311.1316 | Desloratadine | C_19_H_19_ClN_2_ | + | - | - | - | + |
| 179 | 9.47 | 290.1741 | Lycoramine | C_17_H_23_NO_3_ | + | - | - | - | - |
| 180 | 9.47 | 415.1234 | Asperuloside | C_18_H_22_O_11_ | + | - | + | - | + |
| 181 | 9.68 | 263.1283 | Grosheimin | C_15_H_18_O_4_ | + | + | - | - | + |
| 182 | 9.71 | 261.1110 | 8-deoxylactucin | C_15_H_16_O_4_ | + | - | + | - | - |
| 183 | 9.78 | 288.1602 | Piperanine | C_17_H_21_NO_3_ | - | - | - | + | + |
| **Total** | | | **183** |  | **68** | **72** | **39** | **66** | **99** |
