# Supplementary material for: Metabolomic Profiling and Immunomodulatory Activity of a Polyherbal Combination in Cyclophosphamide-Induced Immunosuppressed Mice
Source: Front Pharmacol. 2022 Jan 3;12:647244. doi: 10.3389/fphar.2021.647244 (PMC8762268; doi:10.3389/fphar.2021.647244)
Supplement: Supplementary file 2 [file Presentation1.PPTX]

## Slide 1
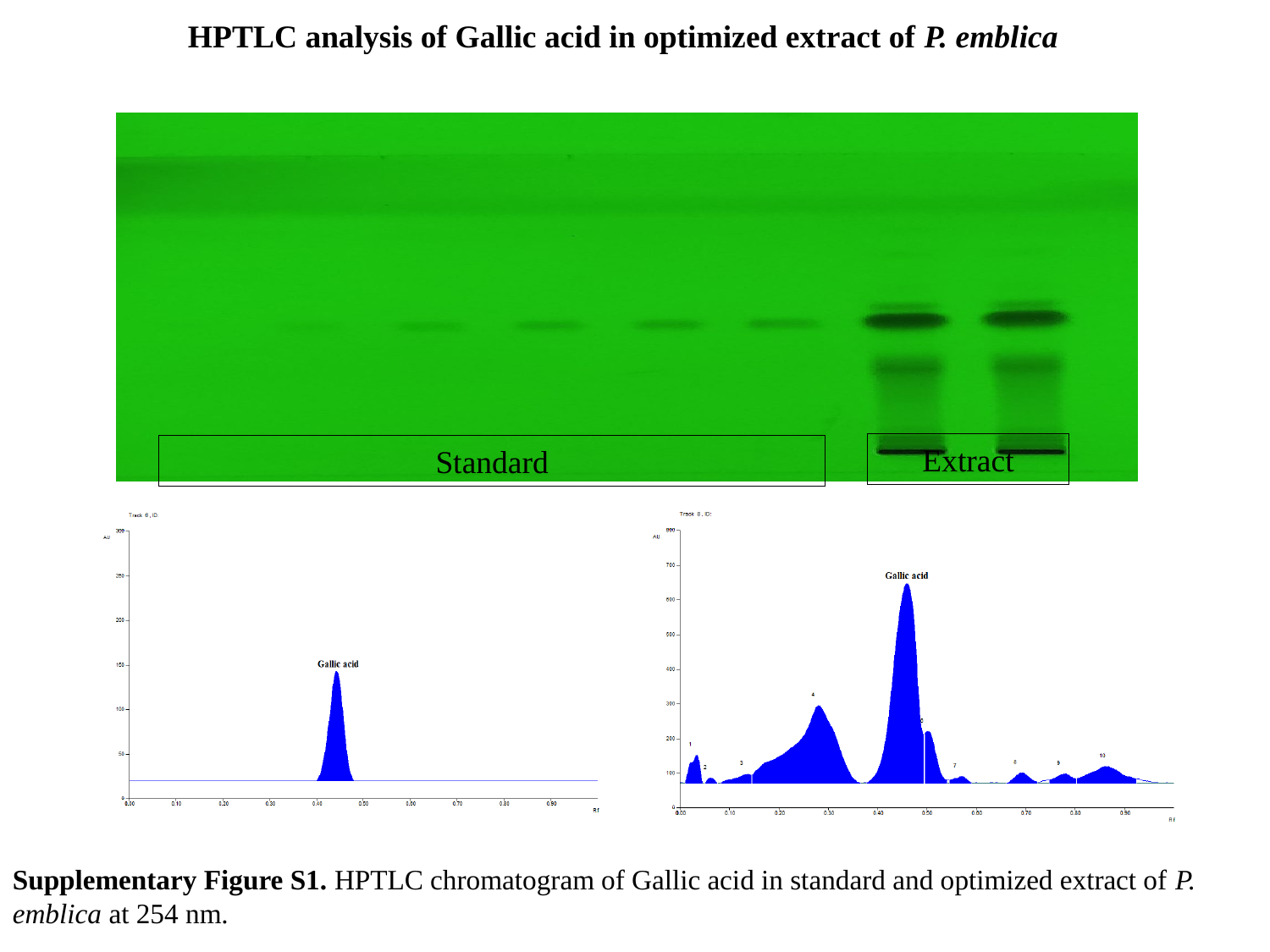

HPTLC analysis of Gallic acid in optimized extract of P. emblica
Extract
Standard
Supplementary Figure S1. HPTLC chromatogram of Gallic acid in standard and optimized extract of P. emblica at 254 nm.

## Slide 2
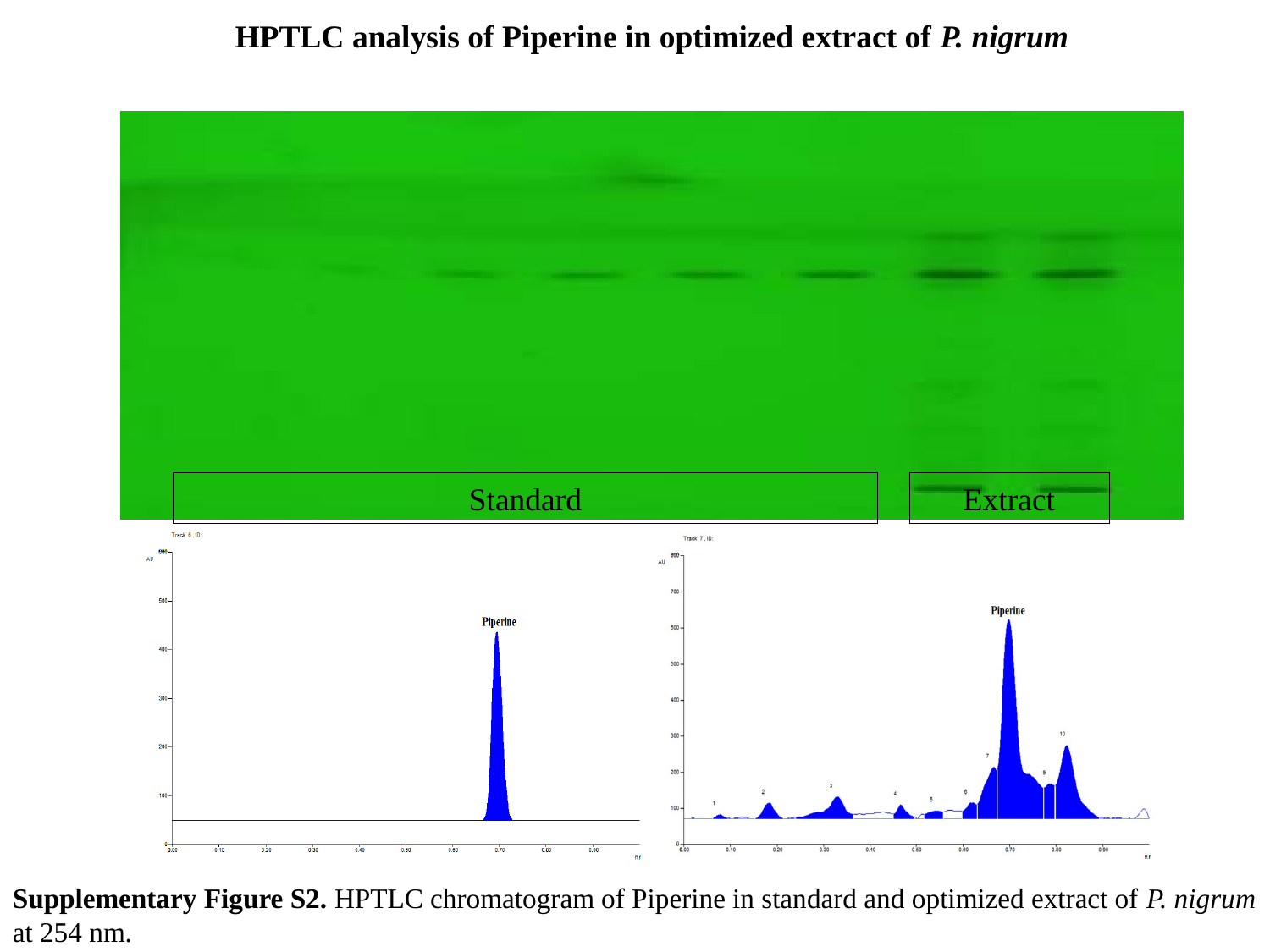

HPTLC analysis of Piperine in optimized extract of P. nigrum
Standard
Extract
Supplementary Figure S2. HPTLC chromatogram of Piperine in standard and optimized extract of P. nigrum at 254 nm.

## Slide 3
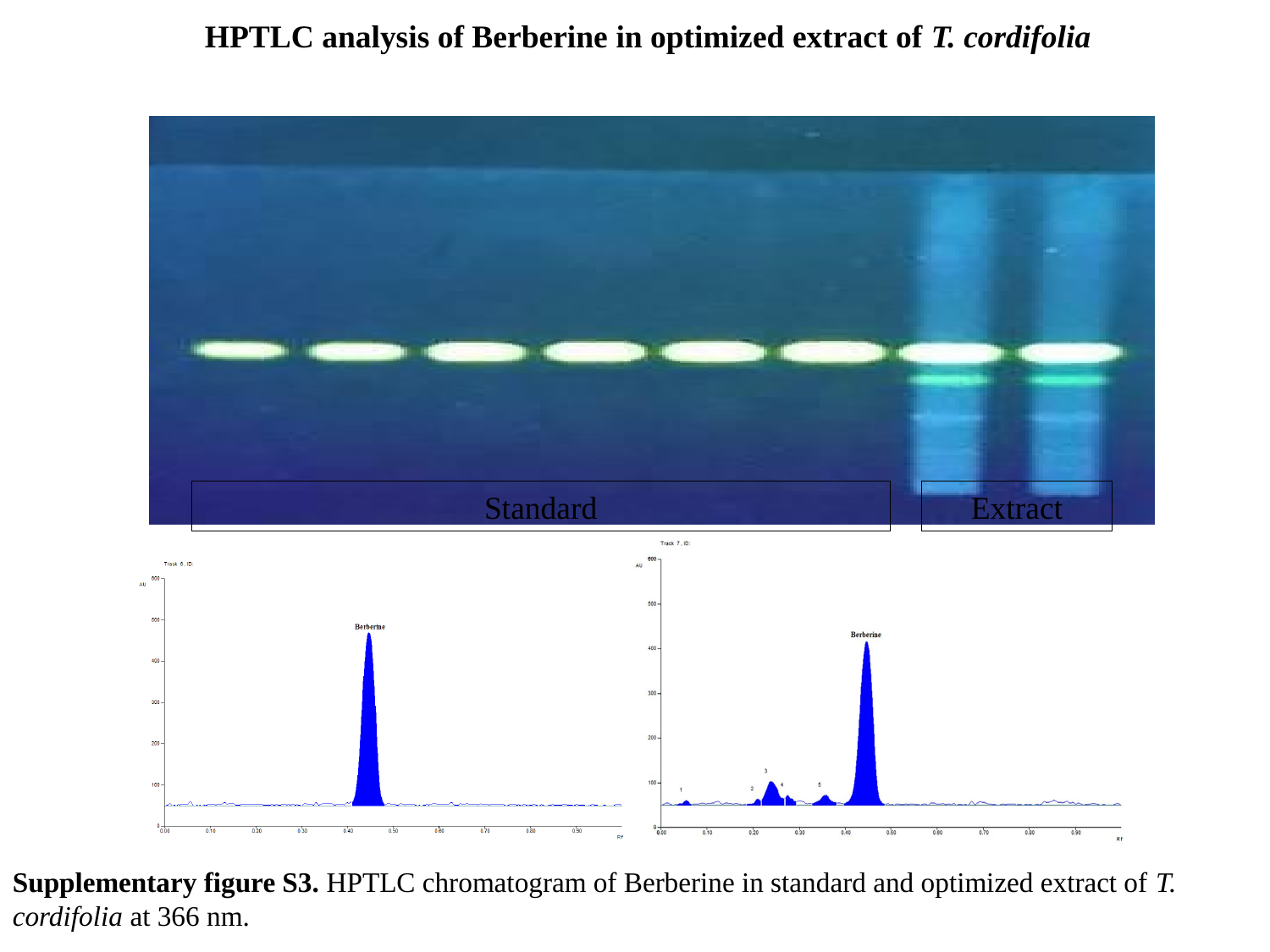

HPTLC analysis of Berberine in optimized extract of T. cordifolia
Standard
Extract
Supplementary figure S3. HPTLC chromatogram of Berberine in standard and optimized extract of T. cordifolia at 366 nm.

## Slide 4
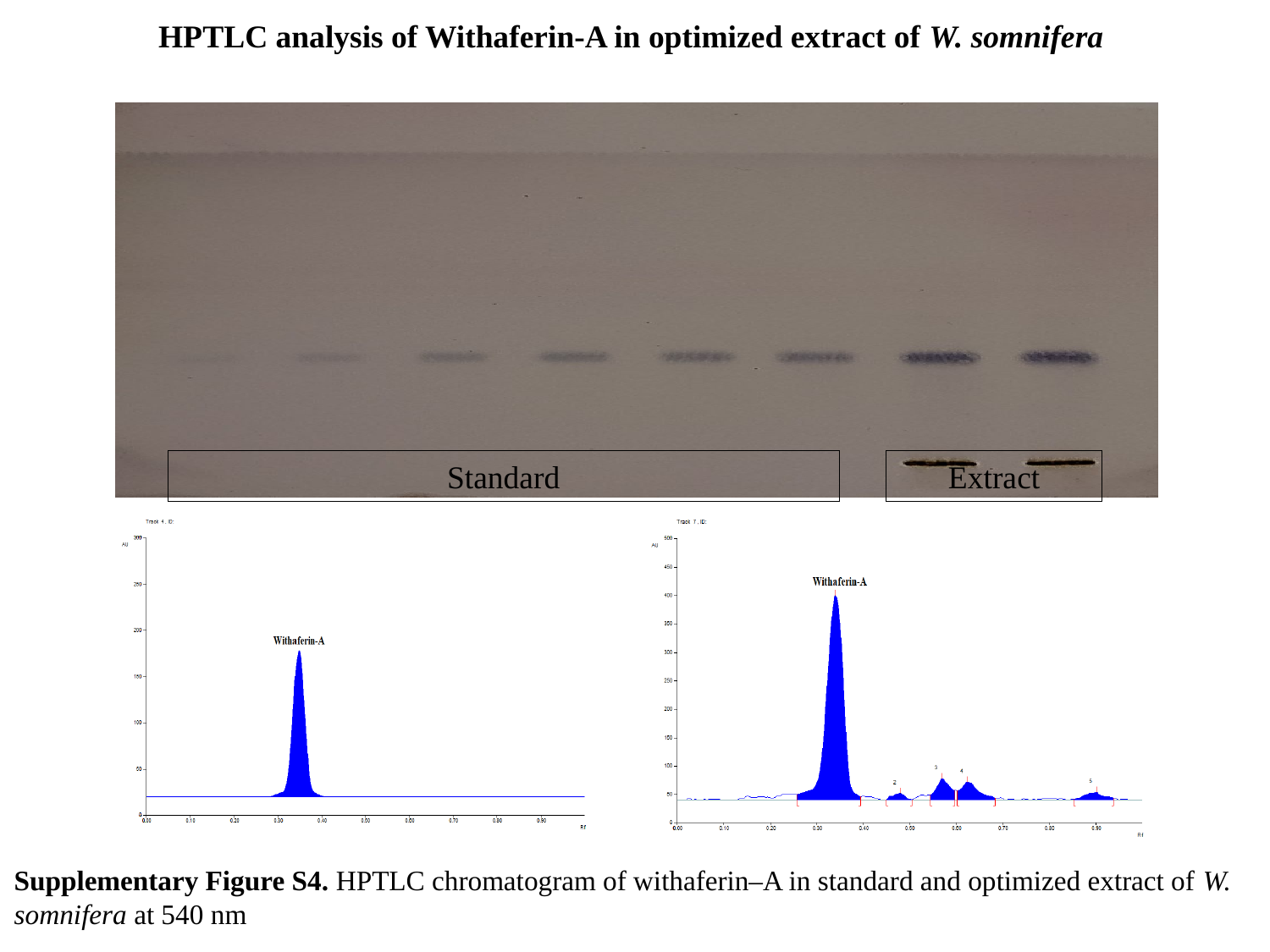

HPTLC analysis of Withaferin-A in optimized extract of W. somnifera
Standard
Extract
Supplementary Figure S4. HPTLC chromatogram of withaferin–A in standard and optimized extract of W. somnifera at 540 nm

## Slide 5
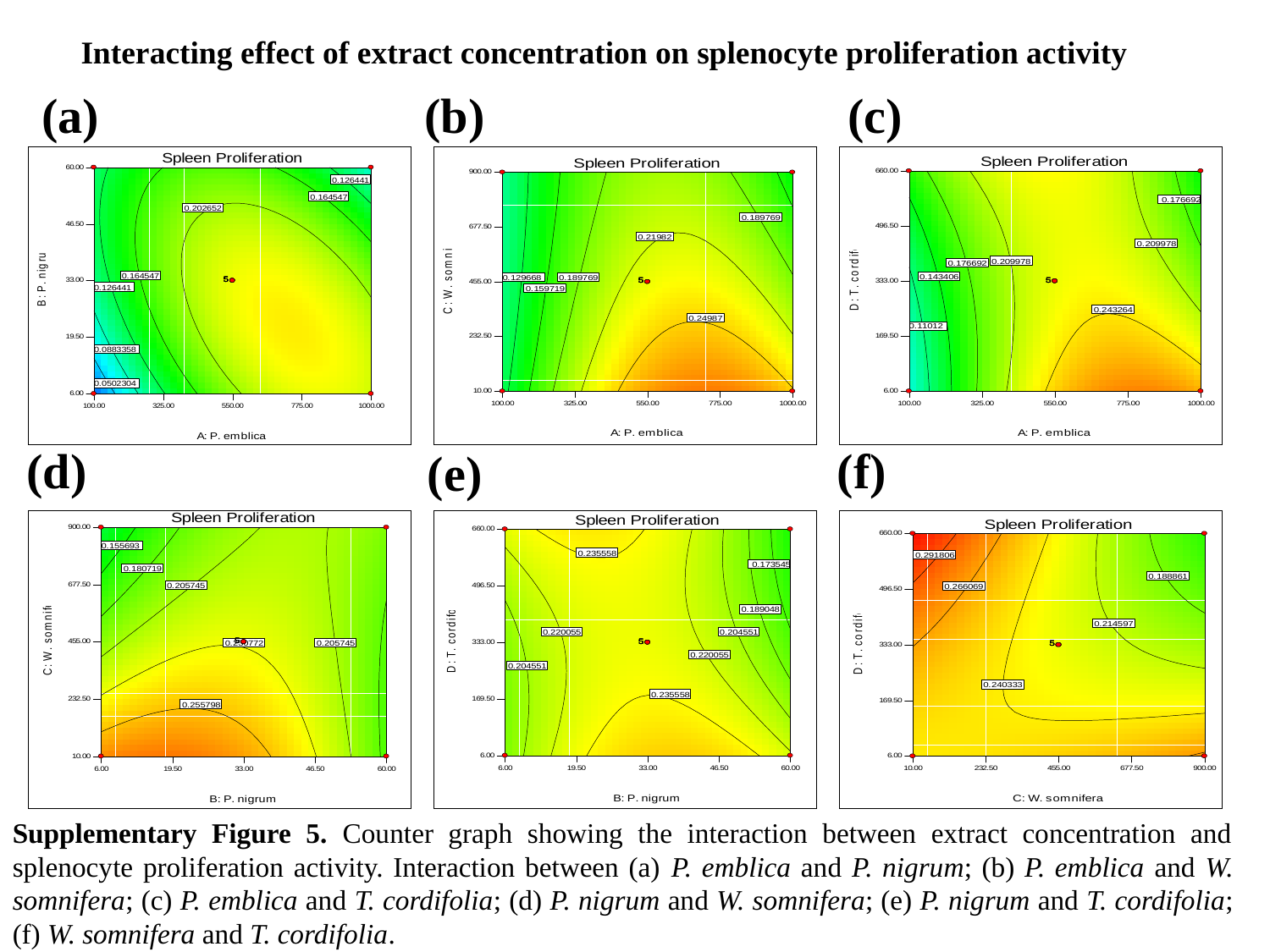

Interacting effect of extract concentration on splenocyte proliferation activity
(a)
(b)
(c)
(d)
(f)
(e)
Supplementary Figure 5. Counter graph showing the interaction between extract concentration and splenocyte proliferation activity. Interaction between (a) P. emblica and P. nigrum; (b) P. emblica and W. somnifera; (c) P. emblica and T. cordifolia; (d) P. nigrum and W. somnifera; (e) P. nigrum and T. cordifolia; (f) W. somnifera and T. cordifolia.

## Slide 6
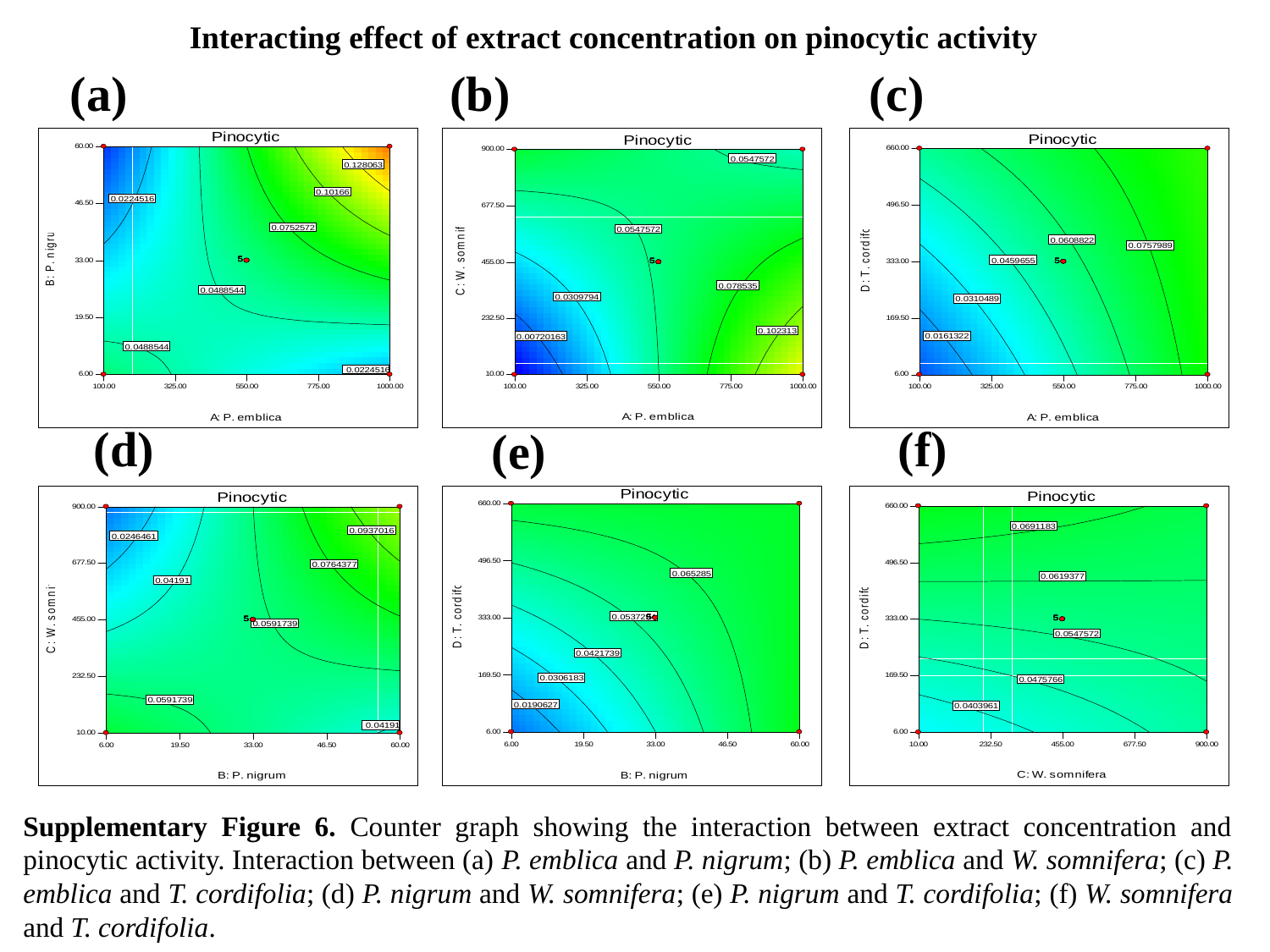

Interacting effect of extract concentration on pinocytic activity
(a)
(b)
(c)
(d)
(f)
(e)
Supplementary Figure 6. Counter graph showing the interaction between extract concentration and pinocytic activity. Interaction between (a) P. emblica and P. nigrum; (b) P. emblica and W. somnifera; (c) P. emblica and T. cordifolia; (d) P. nigrum and W. somnifera; (e) P. nigrum and T. cordifolia; (f) W. somnifera and T. cordifolia.

## Slide 7
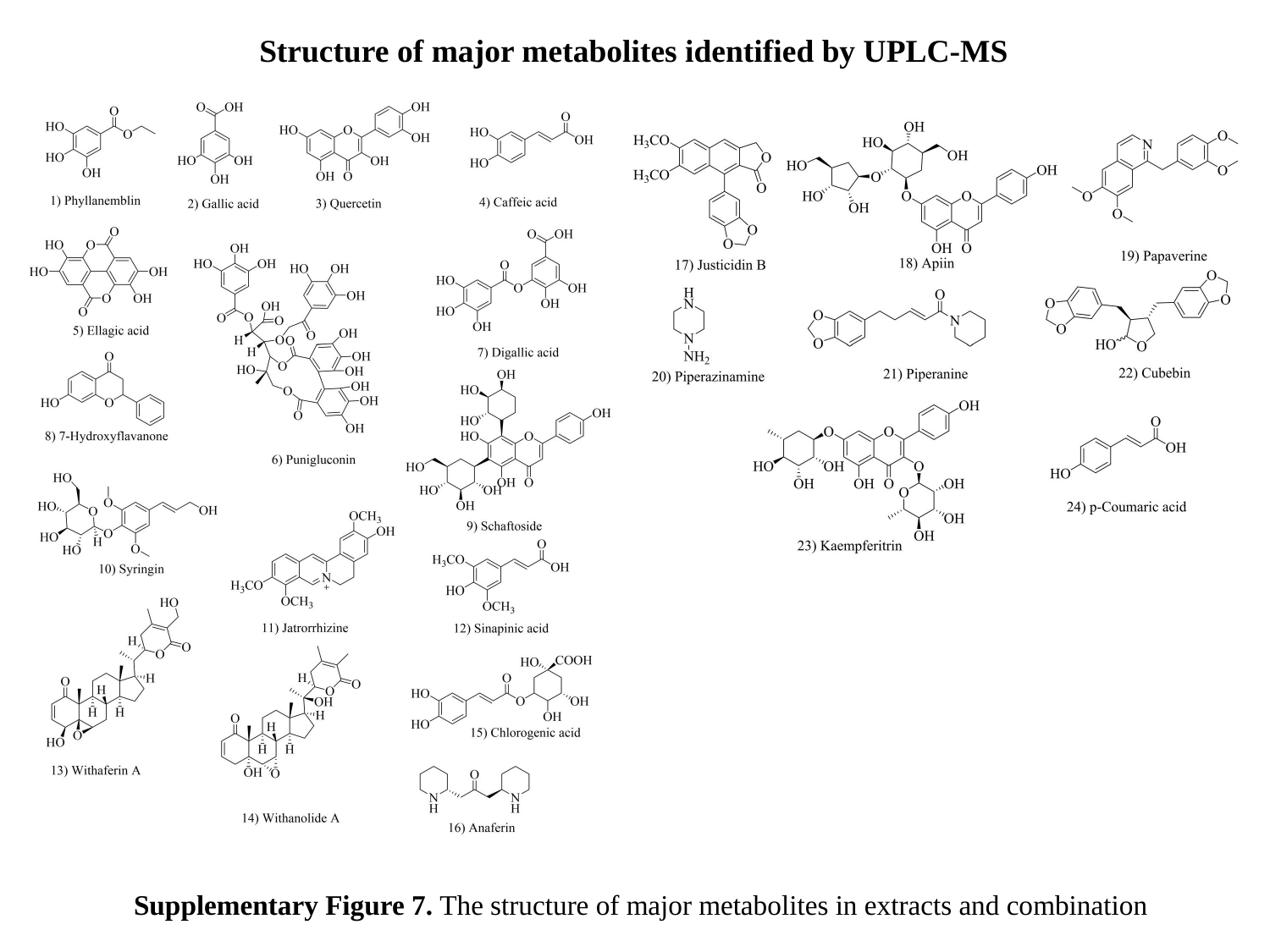

Structure of major metabolites identified by UPLC-MS
Supplementary Figure 7. The structure of major metabolites in extracts and combination

## Slide 8
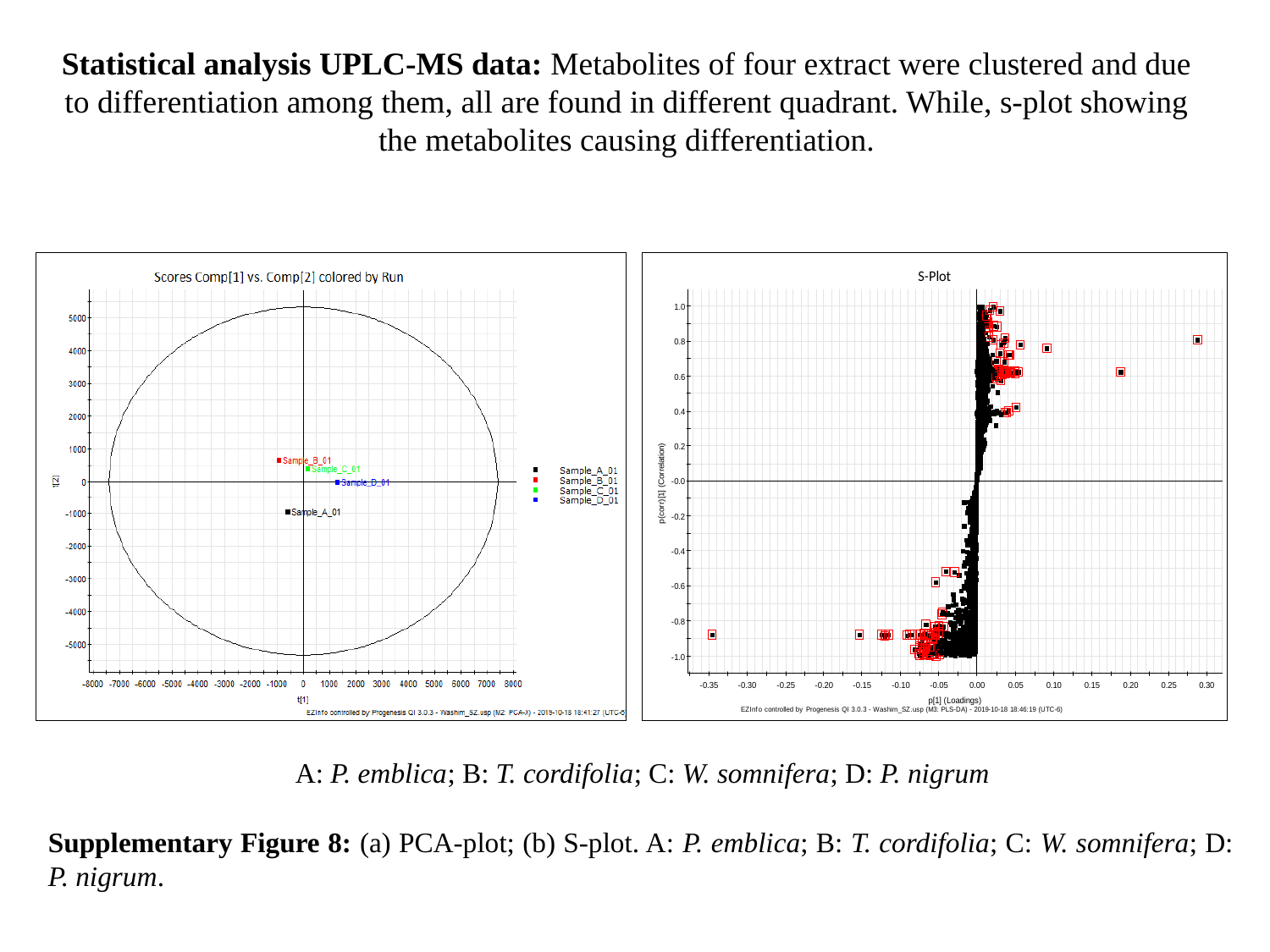

Statistical analysis UPLC-MS data: Metabolites of four extract were clustered and due to differentiation among them, all are found in different quadrant. While, s-plot showing the metabolites causing differentiation.
A: P. emblica; B: T. cordifolia; C: W. somnifera; D: P. nigrum
Supplementary Figure 8: (a) PCA-plot; (b) S-plot. A: P. emblica; B: T. cordifolia; C: W. somnifera; D: P. nigrum.
